# Using Reappraisal to Improve Outcomes for STEM Teachers and Students

**DOI:** 10.5334/joc.313

**Published:** 2023-08-11

**Authors:** Lital Daches Cohen, James J. Gross, Orly Rubinsten

**Affiliations:** 1 Edmond J. Safra Brain Research Center for the Study of Learning Disabilities, University of Haifa, Haifa, Israel; 2 Department of Learning Disabilities, University of Haifa, Haifa, Israel; 3 Department of Psychology, Stanford University, USA

**Keywords:** STEM, Stress, Emotion regulation, Reappraisal

## Abstract

The many stressors associated with teaching can take a toll, resulting in high levels of burnout among teachers and reduced motivation and academic performance among students. This is especially true in the context of science, technology, engineering, and mathematics (STEM) subjects. Despite the efficacy of emotion regulation interventions in pedagogical settings in general and in STEM teaching in particular, there is a lack of suitable interventions. We applied the process model of emotion regulation to STEM teaching and proposed a framework, STEM-Model of EmotioN regulation: Teachers’ Opportunities and Responsibilities (STEM-MENTOR), to elucidate how the high demands of STEM teaching and contextual factors (e.g., culture, reforms, teacher-student interactions) may lead to intensified negative emotions and deficits in executive functioning and emotion regulation implementation. Teacher emotions, in turn, shape students’ STEM-related achievements and epistemic emotions. Thus, teachers’ emotion regulation skills have pervasive effects on teaching outcomes for both teachers and students. We illustrate how at each level of our framework, steps could be taken to improve teachers’ emotional trajectory. Our proposed STEM-MENTOR framework has implications for theoretical understanding and may help to shape future interventions that focus on cognitive-emotional processes in STEM education.

## Introduction

Imagine two classrooms in which the teachers are pressured by parents and school officials to help students achieve the highest levels of academic excellence, while dealing with anxious students facing environmental pressure to achieve high grades. In the first classroom, the teacher is highly stressed, and regularly fails to control her anxiety, and this adversely affects the classroom environment. In the second classroom, the teacher is able to flexibly control her cognitive and emotional state of mind, so that she can act as a calm mentor and create a pleasant and supportive atmosphere.

It is easy to see how both the teachers and students in these two classrooms would have very different outcomes. What is harder to see is which factors determine whether a teacher is more likely to be like the one in the first classroom or the second classroom. Given the stakes, this is a critical question. To date, most research concerned with these sorts of questions has focused on teachers’ academic background (Fung et al., 2017), experience (Brandenburg et.al, 2016), and salary (for a cross-national analysis, see Akiba et al., 2012). Recent studies, however, have highlighted the need to consider other factors (Escalante Mateos et al., 2021), such as the teacher’s affective functioning (Frenzel et al., 2021; Jeon & Ardeleanu, 2020).

In what follows, we consider the unique emotional demands of science, technology, engineering, and mathematics (STEM) teaching in the K-12 classroom. We then present the process model of emotion regulation and discuss the effectiveness of emotion regulation-based intervention in educational settings. Next, we propose an emotion regulation framework for STEM teachers: STEM-Model of EmotioN regulation: Teachers’ Opportunities and Responsibilities (STEM-MENTOR). Our model targets STEM teachers because they may be more sensitive to school environments than non-STEM teachers (Wang et al., 2018), but it may be suitable for a wider population. STEM-MENTOR identifies contextual factors in STEM teaching that can influence the current emotional state of teachers and their decision as to which emotion regulation strategy to use. According to this model, STEM teaching-related stressors create a challenging situation for the implementation of effective emotion regulation. If STEM teachers cannot regulate their emotions, learning processes are impaired, and students adopt biased, inaccurate beliefs that shape their cognitive processing of STEM-related information and events. We conclude by explaining the implications of the STEM-MENTOR framework for theory and interventions that focus on cognitive-emotional processes.

## Emotional Demands of STEM Teaching

Teaching is considered to be an occupation that involves ‘emotional labour’ (Hargreaves, 2000), causing teachers to experience intensified negative emotions, including stress and job dissatisfaction, and diminished psychological well-being (Chan, 2006). Work overload makes it difficult for teachers to focus on teaching (Ramachandran, 2005). Teachers and principals report frequent job-related stress at twice the rate of the general population of working adults (Steiner et al., 2022). Forty-six percent of K-12 teachers in the US report high levels of daily stress (Gallup, 2014; Greenberg et al., 2016), and 61% describe their work as always or often stressful (American Federation of Teachers, 2017). The high stress levels accompanying teaching have profound effects on teachers’ well-being (Buettner et al., 2016) and students’ educational outcomes (Burić, 2019; Harley et al., 2019; Herman et al., 2018).

Three common sources of stress are teachers’ perceptions of unusual working time (i.e., workload stress), students’ misbehavior, and high or unrealistic expectations of authorities and parents with respect to students’ achievement (Collie & Mansfield, 2022). These stressors are particularly relevant in the context of STEM education. STEM subjects are considered stressful to teach (Cui et al., 2018) and learn (Barroso et al., 2021; Becker et al., 2014). Teachers tend to express uncertainty about how to teach STEM courses that have integrated elements such as problem- or project-based learning. They also report uncertainty about how to design STEM courses without losing disciplinary integrity (Estapa & Tank, 2017; Johnson, 2012; Shernoff et al., 2017).

Yet the importance of STEM in Western societies (Hafni et al., 2020), now and in the future (Kaku, 2012), puts these courses at the forefront of education systems. The societal and personal significance (National Research Council, 2014) of STEM professions means STEM students and STEM teachers receive stressful implicit messages from their respective environments. For example, STEM students are pressured to achieve by their parents (Daches Cohen & Rubinsten, 2017). In addition, as the Program for International Student Assessment (PISA) shows, many countries struggle with STEM performance. For example, Canada performs well internationally, but one in six and one in eight Canadian students did not meet the benchmark levels of mathematics and science, respectively (O’Grady et al., 2018). STEM teachers are expected to lead these students to high levels of success while being watched and criticized more than teachers of non-STEM courses (Bybee, 2013). Supporting students’ academic learning has been found to be a top-ranked source of job-related stress for teachers (Steiner et al., 2022), and STEM teachers are particularly affected (Cui et al., 2018). Not surprisingly, the teacher shortage crisis (Holmqvist, 2019) is more pronounced in STEM than non-STEM fields (Cowan et al., 2016).

We argue that teachers’ emotional state matters and emotion regulation skills should therefore be considered a crucial component of teaching opportunities and responsibilities (Braun et al., 2020; Frenzel et al., 2021; Jeon & Ardeleanu, 2020), especially in STEM fields (Bellocchi et al., 2017; Zembylas, 2002).

## Emotion Regulation

There is general consensus that affective and cognitive processes are deeply intertwined (Gardner, 1995), including in STEM learning (Winne, 2019). One particularly important type of affect in this regard is emotion, which is generated when stimuli are meaningful or relevant to the individual, attract his/her attention, and are evaluated in relation to valued goals (Gross, 2015), suggesting the central role of appraisals in generating and shaping emotion (Conte et al., 2022). According to the appraisal-driven componential approach (Sander et al., 2018), five complementary and interrelated brain networks comprise the emotional brain: elicitation, expression, autonomic reaction, action tendency, and feeling. Emotion elicitation is dependent on an appraisal process, while the other components are generally considered to reflect the emotional response.

Emotional states depend on how the individual evaluates the situation (i.e., cognitive appraisal; Lazarus, 2001), and emotion regulation refers to whether and how the individual attempts to change an appraisal (Yih et al., 2019). Emotion regulation includes an array of processes (Rottenberg & Gross, 2003) by which the individual influences the type, intensity, duration, and expression of both positive and negative emotions (Gross, 2015). In the classroom, teachers tend to express positive emotions (Taxer & Frenzel, 2015) and are confident in their ability to communicate and up-regulate these emotions (Sutton et al., 2009). Compared to this, they are less confident in their ability to down-regulate their negative emotions (Sutton et al., 2009), although most of their regulation attempts are to down-regulate negative emotions by trying to hide them, while simultaneously faking positive emotions (Taxer & Frenzel, 2015; Taxer & Gross, 2018).

**Figure 1:**
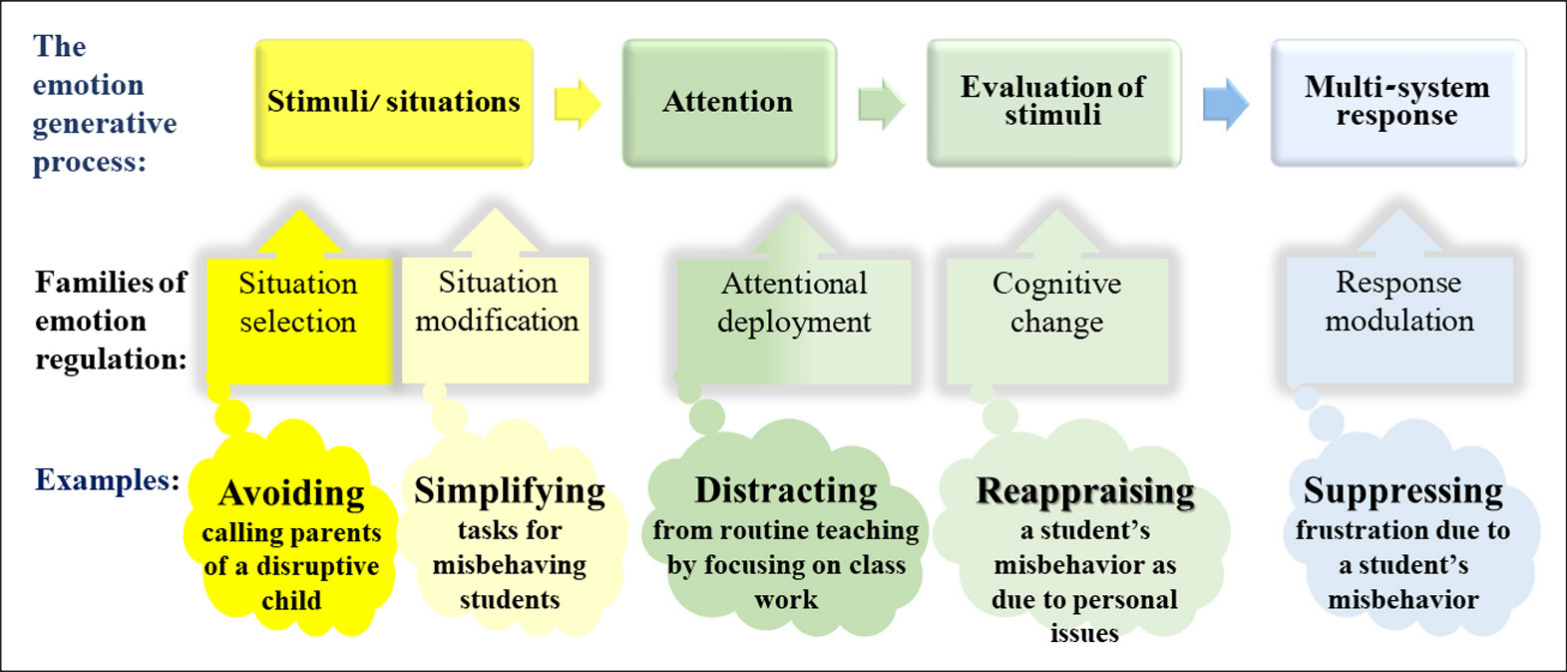
Process model of emotion regulation (Gross, 2015) applied to a classroom context.

In the process model of emotion regulation, Gross (2015) distinguishes between regulatory strategies (Figure 1, second row) at various stages of the emotion generative process (Figure 1, first row). Two widely studied emotion regulation strategies are *cognitive reappraisal* and *expressive suppression* (Bigman et al., 2017; Gross, 2015). Cognitive reappraisal constitutes an antecedent-focused strategy that involves the adoption of an objective perspective (reappraisal as rethinking; e.g., Sheppes & Meiran, 2008) or active attempts to adopt a positive perspective (reappraisal as reframing; e.g., Ochsner et al., 2002) in order to change the way the situation has been appraised (Gross, 2015). In contrast, expressive suppression is a response-focused strategy in which the individual attempts to conceal feelings, behaviors, and physiological activity (Gross, 2015).

Difficulty regulating emotional states, or emotion dysregulation (Gross, in press) can manifest in emotion regulation failure (e.g., Kwon et al., 2018; Pizzie & Kraemer, 2017), emotion misregulation that does not well match the situation, and emotion regulation misexecution (e.g., Harley et al., 2019; Pizzie & Kraemer, 2021; Skaalvik, 2018; Xu et al., 2019). Drawing on the documented costs and benefits of different emotion regulation strategies in the process model of emotion regulation (Gross, 2013; Sheppes et al., 2015), each of these types of emotion dysregulation can be analyzed (Gross, in press).

At the *intra-personal* level, reappraisal has been found generally effective in reducing subjective negative affect (Balzarotti et al., 2017; Goldin et al., 2019; Gross, 2013, 2015; Troy et al., 2018) in math-related situations (e.g., Jamieson et al., 2016; Pizzie & Kraemer, 2021) and leads to more adaptive behavioral (Balzarotti et al., 2017; McRae, Ciesielski, et al., 2012; Schönfelder et al., 2014), physiological (Liu et al., 2019; McRae, Ciesielski, et al., 2012; Sammy et al., 2017), and neural responses to emotionally evocative events (Goldin et al., 2019; Schönfelder et al., 2014). Suppressing emotions is less effective than reappraisal (Gross, 2013, 2015) and is linked to increased strain and emotional exhaustion among teachers (Burić et al., 2021; Chang, 2013; Hülsheger et al., 2010; Taxer & Frenzel, 2015) and lower quality of instruction (Burić & Frenzel, 2021). At the *inter-personal* level (Zaki & Williams, 2013), teachers’ emotion regulation tendencies are thought to influence teacher-student interactions (Braun et al., 2019) and teachers’ supportive reactions to students’ emotions (Jeon et al., 2016; Swartz & McElwain, 2012). Moreover, by modeling effective emotion regulation, teachers can impact their students’ emotion regulation tendencies (Brady et al., 2018; Fried, 2011; Harley et al., 2019).

To effectively regulate emotion, the individual must be aware of the emotion and the relevant context, know and activate his/her emotion-regulatory goals, and make a skillful choice and implementation of an emotion regulation strategy while protecting the emotion-regulatory goal and adjusting it when/if the situation changes (Gross, 2013; Gross & Jazaieri, 2014). People may implement multiple regulatory strategies in a given emotional episode (Aldao & Nolen-Hoeksema, 2013), a phenomenon recently termed emotion polyregulation (Ford et al., 2019). It is not simply the depth of the emotion regulation repertoire that matters (Bonanno & Burton, 2013). The specific class of strategies included in the repertoire (Grommisch et al., 2019; Southward & Cheavens, 2020) and the ability to choose strategies synchronized with contextual demands and personal goals (emotion regulation flexibility; Aldao et al., 2015) also matter (Greenaway et al., 2018; Wilms et al., 2020). For example, choosing a less effortful distraction strategy has been associated with adaptive functioning among young children with low, but not high, working memory (Dorman Ilan et al., 2019). In this vein, there are times when it may be beneficial not to regulate negative emotions but to express them genuinely, due to the important role they may serve, such as guiding appropriate behavior and motivating the individual to improve his/her circumstances (Feinberg et al., 2020; Ford & Troy 2019). For instance, the use of reappraisal to control guilt and shame results in increased job satisfaction and decreased burnout, but also increased counterproductive workplace behaviors (Feinberg et al., 2020).

## Reappraisal-Based Interventions in Educational Settings

There is growing interest in emotion regulation interventions in pedagogical settings in general and in STEM contexts in particular. Two types of these interventions can be found in the literature, one aimed at changing the interpretation of an emotional event (i.e., situation-focused; Yeager & Dweck, 2020) and the other targeting the response appraisals (i.e., response-focused; Jamieson et al., 2013). An example of event-focused intervention is the growth mindset (Ford & Gross, 2019; Yeager & Dweck, 2020) which teaches people that ability is not fixed but can be developed with effort, effective strategies, and support, leading to a reinterpretation of challenges as facilitators of personal development and controllable (Yeager et al., 2022). Three different large studies have replicated the efficacy of such interventions in improving achievements for low-achieving adolescents and increasing participation rate in harder math classes (Rege et al., 2021; Yeager et al., 2016, 2019). Response-focused interventions target the interpretation of arousal as a functional resource for psychological, biological, and behavioral outcomes (Jamieson et al., 2010, 2016; John-Henderson et al., 2015; Sammy et al., 2017). This line of research has demonstrated that teaching people about the adaptive benefits of stress arousal before a standardized test directly reduces adults’ and adolescents’ acute stress responses (e.g., math anxiety; Jamieson et al., 2016; Pizzie & Kraemer, 2021) and improves performance (e.g., Brooks, 2014; Jamieson et al., 2010; Pizzie et al., 2020; Rozek et al., 2019; but see Ganley et al., 2021).

In more recent work (Yeager et al., 2022), a self-administered online training module integrated the two main types of reappraisal manipulations by suggesting difficult challenges should be perceived as valuable opportunities for self-improvement (i.e., situation-focused), and physiological stress responses can fuel optimal performance (i.e., response-focused). This intervention showed a high level of promise in reducing evaluative stress and stress-related physiological responses and increasing psychological well-being among secondary and post-secondary students. Although these intervention studies did not focus on teachers, they suggest that reappraisal-based interventions may help teachers reappraise their view of stressful situations. Research has indicated the benefits of teachers› use of reappraisal, including in managing the stress associated with classroom activities (Chang & Taxer 2021; Jiang et al., 2016; Katz et al., 2018; Lee et al., 2016) and the overall teaching experience (Braun et al., 2019; Chatzistamatiou et al., 2014; Feldman & Freitas, 2021; Jeon et al., 2016; Lee et al., 2016; Moè & Katz, 2020; Oberle & Schonert-Reichl, 2016; Russo et al., 2020; Sutton & Harper, 2009; Swartz & McElwain, 2012; Yan et al., 2011). Emotion regulation interventions may change the ‘cognitive story’ that teachers tell themselves in relation to their experiences of stress and pressure, improving the way they deal with stressors in the future (Rozek et al., 2019). Stress reappraisal interventions may be particularly suitable for STEM teachers because they may experience increased stress at work (Schleicher, 2019) and often report intensified negative emotions (Cowan et al., 2016; Cui et al., 2018; Hembree, 1990).

A number of therapeutic approaches incorporate emotion regulation training to encourage awareness of emotions and the use of more adaptive emotion regulation strategies, including emotion regulation therapy (Mennin & Fresco, 2009) and acceptance of emotional responses (Roemer et al., 2008). These interventions can reduce teachers’ emotional dissonance between the experienced emotion and the emotional expression (Keller et al., 2014). This dissonance can be detrimental to teachers’ occupational well-being (Näring et al., 2006) and students’ emotions (Keller & Becker, 2021). Roeser et al. (2012) reviewed mindfulness training for teachers that included explicit instructions on emotions and stress and explained how to regulate them more effectively using mindfulness. An example is Cultivating Awareness and Resilience in Education (CARE; Jennings et al., 2019). Elementary school teachers who participated in mindfulness-based professional development through CARE reported both sustained and new benefits in well-being at a follow-up assessment almost one-year post-intervention compared to teachers in a control group.

Given the high stress levels accompanying the teaching (Cui et al., 2018) and learning (Barroso et al., 2021; Becker et al., 2014) of STEM subjects, the dearth of research-based interventions that incorporate aspects of reappraisal in educational settings is surprising. Most existing interventions were designed for students in STEM fields. These interventions have been found to have positive emotional (e.g., Jamieson et al., 2016; Pizzie & Kraemer, 2021) and academic outcomes (e.g., Brooks, 2014; Pizzie et al., 2020; Rege et al., 2021; Yeager et al., 2016, 2019).

## STEM-MENTOR Model

**Figure 2:**
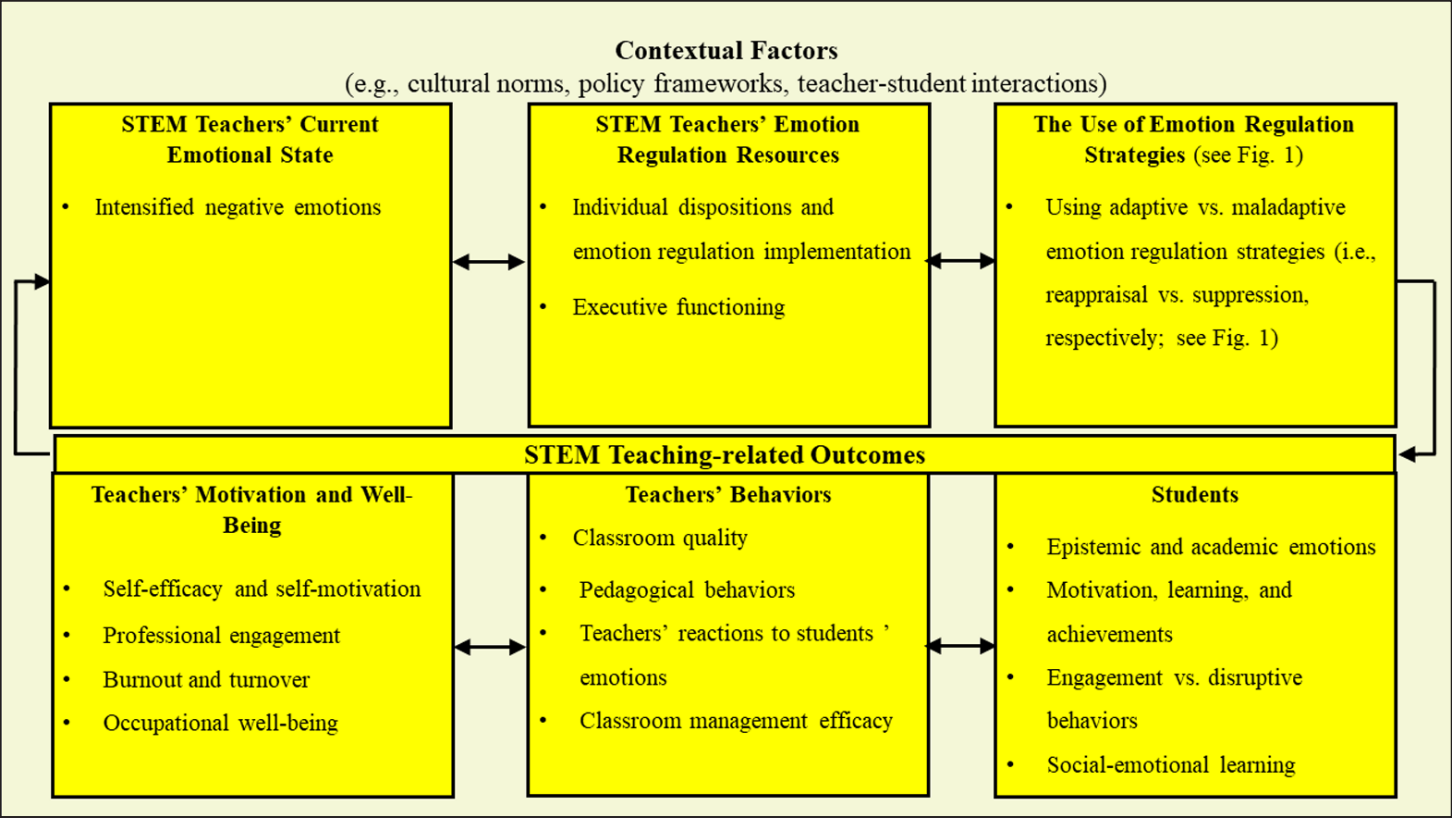
STEM-Model of EmotioN Regulation (STEM-MENTOR).

We argue that emotion regulation-based interventions targeting STEM teachers’ emotion regulation skills have the potential to create an emotionally supportive atmosphere in the classroom. Developing STEM teachers’ knowledge of and ability to regulate their own emotions will, in turn, help students develop regulatory skills and succeed academically (Denham et al., 2012; Taxer & Gross, 2018). Applying the process model of emotion regulation (Gross, 2015; Figure 1) to STEM teaching, we propose a framework (see Figure 2) suggesting that STEM teachers’ emotions and emotion (dys)regulation may have profound effects on their students’ cognitive processing of STEM-related information. Specifically, STEM teachers’ emotional expressions constitute top-down environmental-emotional knowledge that extends beyond the teachers to influence their students’ emotions and behavior. By modeling, teachers can affect the development of students’ emotion regulation tendencies (Brady et al., 2018; Fried, 2011; Harley et al., 2019; Zaki & Williams, 2013).

As shown in Figure 2, STEM teachers’ current negative emotional state (upper left square) disrupts their emotion regulation resources (upper middle square), leading to the use of maladaptive (vs. adaptive) emotion regulation strategies (upper right square). The broader context can also influence the teachers’ current emotional state and their decision as to which emotion regulation strategy to use (the external frame). The use of a maladaptive emotion regulation strategy negatively affects the teachers’ motivation and well-being (bottom left square), and this, in turn, creates a challenging situation for the implementation of effective STEM teaching-related behaviors (bottom middle square). The emotional experiences (via STEM environment) and emotional expressions (via emotion regulation) of STEM teachers also shape students’ STEM-related emotions and behaviors (bottom right square). In the following sections, we elaborate on each of these in turn.

### Contextual Factors (External Frame)

An integrative affect-regulation framework (Troy et al., 2022) highlights the role of contextual features in the use of emotion regulation strategies, their short-term consequences, and the relationships between emotion regulation strategies and resilience. These contextual features include characteristics of the negative situation, such as intensity (Bonanno et al. 2015), controllability (Haines et al. 2016; Troy et al. 2013), timing, and duration (Epel et al. 2018). For example, reappraisal has been linked to increased resilience in relatively uncontrollable adversity but not in relatively controllable adversity (Haines et al. 2016; Troy et al. 2013) because reappraising controllable situations may decrease one’s motivation to change it (Ford & Troy 2019).

Broader aspects, such as one’s culture, have also been recognized as playing a key role in the use of emotion regulation strategies (Markus & Kitayama 1991; Mesquita 2001). For example, a meta-analysis revealed cultural differences in the effect of suppression on resilience (Hu et al. 2014), possibly due to the higher value of suppressing negative emotions in collectivistic-oriented cultures, such as Asian cultures, compared to more individualistic-oriented cultures, such as European ones (Markus & Kitayama 1991; Mesquita 2001).

Cultural norms on emotions related to STEM (Foley et al., 2017; Stoet et al., 2016) and their expression (Stoet et al., 2016) are especially relevant to our proposed theoretical framework. For example, math anxiety tends to be more prevalent in Asian countries than Western European countries (Lee, 2009). In addition, economically-developed and gender-equal countries show a lower prevalence of math anxiety (Stoet et al., 2016). Cultural norms also shape emotional display rules guiding teachers’ decisions either consciously or unconsciously about the appropriate expression of emotions in the classroom (Butler et al., 2007; Ford & Gross, 2019; Ford & Mauss, 2015; Hagenauer et al., 2016; Schutz et al., 2009). To promote a positive learning environment, for example, teachers are expected to display enthusiasm and caring behaviors in the classroom. In two studies, teachers who endorsed display rules were more likely to use suppression as their habitual way to regulate emotions (Chang, 2020; Taxer & Frenzel, 2015). A recent meta-analysis (Stark & Bettini, 2021) found teachers perceived emotional display rules as promoting their emotional support of students, contributing to their professional development, and fostering students’ academic development. Two-thirds of the teachers in Sutton et al.’s (2009) study reported less teaching effectiveness after expressing negative emotions, possibly leading them to suppress their negative emotions in the classroom.

Larger policy frameworks and school contexts may also affect teachers’ emotions (Jiang et al., 2021; Richardson & Watt, 2018). STEM teachers have experienced a stream of educational changes and reforms (Li et al., 2020; Stehle & Peters-Burton, 2019), and their emotions can be strongly affected by these changes (Jiang et al., 2021; Lee et al., 2013; Tsang & Kwong, 2017). If educational changes and reforms do not support their professional needs, goals, and development, they may have negative feelings (Jiang et al., 2021; Tsang & Kwong, 2017), and this emotional state, in turn, may have significant implications for teacher-student interactions (Aldrup et al., 2018; Braun et al., 2019; Collie et al., 2012; Frenzel et al., 2016; Whitaker et al., 2015). The quality of the teacher-student relationship has been found at affect students’ emotions (e.g., Jamal et al., 2013) and their academic performance (e.g., Malmberg & Hagger, 2009).

Yet teachers have a basic need for relatedness with their students (Spilt et al., 2011). Thus, the emotions of teachers and their relations with their students have effects on teachers’ physical and psychological well-being and also on their professional engagement, burnout, and turnover (Buettner et al., 2016; Harding et al., 2019; Montgomery & Rupp, 2005; Steinhardt et al., 2011; Taxer & Frenzel, 2015). Good relationships with students can have positive emotional impacts on teachers (Aldrup et al., 2018; Veldman et al., 2013), while negative relationships have been associated with negative affective outcomes (Gastaldi et al., 2014; Hagenauer et al., 2015). From the perspective of the transactional model of stress and coping (Lazarus & Folkman, 1984), the failure to establish a caring relationship with students leads to a negative emotional experience for the teacher because this goal is inherent to the teaching profession (Butler, 2012) and is at the core of teachers’ professional identity (van der Want et al., 2015).

### STEM Teachers’ Current Emotional State (Top Left Box)

As a result of the contextual factors and the environmental pressures to achieve in STEM (Cui et al., 2018), academic anxiety is common in STEM fields (e.g., math anxiety) (Hart & Ganley, 2019), extending beyond the general population (Beilock & Willingham, 2014; Foley et al., 2017) to include those learning and teaching math (Ganley et al., 2019; Hembree, 1990). For example, students studying early education have higher levels of math anxiety than those in other fields of study (Hembree, 1990), possibly because of the minimal mathematics requirements to major in elementary education (Malzahn, 2013). However, it is likely that other teachers also experience math anxiety. Most teachers are women (Beilock et al., 2010), and women often are more emotionally reactive to numerical information (Daches Cohen et al., 2021) and display greater academic anxiety in STEM fields than men (Devine et al., 2018; Hart & Ganley, 2019). In general, math-related stimuli are associated with negative emotional reactions (Daches Cohen et al., 2021; Hart & Ganley, 2019). For example, exposure to math-related stimuli, like exposure to negative stimuli, leads to delayed and increased pupil dilation compared to neutral valence stimuli (Layzer Yavin et al., 2022). These findings hint at both the cognitive effort (Shechter & Share, 2021) and the emotional effort (e.g., pleasant and unpleasant stimuli, Bradley et al., 2008) required when exposed to math-related stimuli.

Teachers’ math anxiety has been found to impact their teaching self-efficacy (Gresham, 2008), confidence (Bursal & Paznokas, 2006), and pedagogical behavior (Ramirez et al., 2018), with a concomitant effect on their students’ achievements (Beilock et al., 2010; Ramirez et al., 2018; Schaeffer et al., 2021) and learning regardless of the educational level (Beilock et al., 2010; Schaeffer et al., 2021). The tendency of high math-anxious individuals to avoid math activities (Choe et al., 2019; Hembree, 1990) may cause high math-anxious teachers to provide lower quality math instruction (Akinsola, 2008) and to have lower expectations of their students, either explicitly or implicitly, than lower math-anxious teachers (Ramirez et al., 2018).

### STEM Teachers’ Emotion Regulation Resources (Top Middle Box)

Under the transactional model of stress and coping (Lazarus & Folkman, 1984), teachers’ perceived emotions can be seen as not just a function of exposure to environmental factors, but also as a function of their ability to handle these emotions. Emotional stimuli capture attention; thus, cognitive effort is required (Banich et al., 2009). High executive functions, a set of higher cognitive processes that enable planning, forethought, and goal-directed behavior (Daches Cohen & Rubinsten, 2022), may serve as a protective factor for STEM teachers’ stress (Friedman-Krauss et al., 2014), enabling them to use adaptive emotion regulation strategies in response to emotionally arousing events (Cohen & Mor, 2018; McRae, Jacobs, et al., 2012; Quinn & Joormann, 2020; Wante et al., 2017). For example, neuroimaging studies found reappraisal was associated with the activation of the fronto-cingular network (Buhle et al., 2014) which is involved in domain-general executive control (Niendam et al., 2012). Against this background, several researchers found reappraisal ability (McRae, Jacobs, et al., 2012; Quinn & Joormann, 2020; Toh & Yang, 2022) and frequency (Wante et al., 2017) were linked to executive control of attention. Importantly, Toh and Yang (2022) found a significant link between common executive functions and reappraisal, even when covariates were controlled for, including intelligence, gender, depressive symptoms, age, and social desirability. Hence, the link between executive function and reappraisal seems to be a replicable phenomenon.

In a study relevant to our proposed framework, Daches Cohen and Rubinsten (2022) investigated the relations between math anxiety, emotion regulation, and executive control of attention using multiple two-stage hierarchical linear regression models. Their analyses indicated that the ability of math-anxious university students to use reappraisal in daily life was associated with their ability to avoid heightened emotional reactions when encountering math-related (i.e., threatening) but not negative (i.e., emotional distractions induced by irrelevant words with negative valence) information when executive control of attention was required (i.e., incongruent trials). It bears noting that the contribution of suppression to the regression model was not significant. Indeed, intervention studies have suggested that training in executive control of attention can lead to reduced use of maladaptive regulatory strategies, such as rumination (Cohen et al., 2015; Daches & Mor, 2014), and higher and better implementation of an adaptive reappraisal strategy (Cohen & Mor, 2018).

However, executive functioning is also generally thought to be impaired by stress (Shansky & Lipps, 2013; Shields et al., 2016; Uribe-Mariño et al., 2016), burnout (Deligkaris et al., 2014), and emotional fatigue (Grillon et al., 2015). For example, among preschool teachers, higher executive function skills were related to lower job stress (Friedman-Krauss et al., 2014). Stress affects multiple biological processes with known effects on executive functions, such as catecholaminergic activity and corticotropin-releasing hormone (Shansky & Lipps, 2013; Uribe-Mariño et al., 2016). Psychological factors may contribute to the effects of stress on executive function as well (Shields et al., 2016). For example, negative social evaluation associated with common stressors may result in rumination on perceived poor performance (De Lissnyder et al., 2012), leading to reduced executive control (Philippot & Brutoux, 2008).

Teachers’ emotions are also determined by individual dispositions (Schutz et al., 2006). Some STEM teachers may initially use maladaptive emotion regulation strategies (see Figure 1) as a core maladaptive skill. The literature has suggested specific neurobiological markers for the use of reappraisal, such as decreased resting-state functional connectivity (rs-FC) between the Middle Temporal Gyrus and occipito-parietal regions and between prefrontal and occipito-parietal brain regions and microstructural anomalies across white matter tracts connecting temporal, parietal, and occipital brain regions (Vitolo et al., 2022). In another line of research, personality traits such as low urgency and high distress intolerance, were linked to disengagement emotion regulation strategies (e.g., suppression) when exposed to high arousal negative affect (Sandel-Fernandez et al., 2022).

Some STEM teachers may have initial or core adaptive emotion regulation skills, but the stress (Shields et al., 2016; Vogel et al., 2016) and burnout (Golkar et al., 2014) in STEM teaching (Cowan et al., 2016; Cui et al., 2018) may disrupt their typical prefrontal cortical function, thus interfering with the successful execution of emotion regulation (Ochsner et al., 2012). For instance, in a sample of elementary and high-school teachers, negative emotions decreased the teachers’ reappraisal and coping abilities (Chang, 2013). These findings are consistent with previous research identifying the tendency to reappraise relatively low-intensity stimuli and to distract oneself from relatively high-intensity stimuli (e.g., Dixon-Gordon et al., 2015; Levy-Gigi et al., 2016; Shafir et al., 2015, 2016; Sheppes et al., 2011, 2014; Wilms et al. 2020). Compared to attentionally engaging and appraising emotional information (i.e., reappraisal), attentional disengagement from emotional information may be effective in modulating high-intensity emotions while requiring minimal cognitive resources (Sheppes, 2020).

In both cases, the environmental or contextual factors related to the STEM profession (i.e., stress and negative emotions) may affect STEM teachers’ motivation to regulate emotions (Yin et al., 2018). The motivation for emotion regulation can be hedonic or instrumental; the former includes approach and avoidance motivations to change the immediate phenomenology of emotion (Tamir, 2016; Tamir & Millgram, 2017), and the latter targets potential consequences of the desired emotional state (e.g., Tamir et al., 2015). Teachers’ hedonic goals for regulating emotions mainly focus on reducing the intensity of their own (intrinsic regulation functions) or their students’ (extrinsic regulation functions) experienced or expressed negative emotions (Taxer & Gross, 2018; Uzuntiryaki-Kondakci et al., 2022). Their instrumental goals are to increase teaching effectiveness and professionalism and manage students’ misbehavior (Gong et al., 2013; Sutton, 2004; Sutton et al., 2009; Taxer & Gross, 2018).

### Use of Emotion Regulation Strategies (Top Right Box)

In the classroom, teachers use emotion regulation on a daily basis and even from lesson to lesson (Keller et al., 2014). They may have a wide repertoire of emotion regulation strategies (Chang & Taxer, 2021; Taxer & Gross, 2018), but findings show they most frequently use suppression (Gong et al., 2013; Taxer & Gross, 2018) and hide their negative emotions in classroom situations (Keller et al., 2014; Taxer & Frenzel, 2015). For example, teachers may use suppression to hide their negative emotions in response to students’ behavioral problems (Jeon & Ardeleanu, 2020; Jeon et al., 2016; Taxer & Gross, 2018).

Although suppression can be an effective form of emotion regulation for managing the classroom environment (Jeon & Ardeleanu, 2020), studies show teachers who use more suppression are less likely to have social support and more likely to have difficulty connecting emotionally with students (Gross, 2013, 2015) in the everyday school context (Jiang et al., 2016), and this could contribute to more negative emotions (Gross, 2002; Jiang et al., 2016; Lee et al., 2016). In addition, the use of suppression requires the individual to control the emotional expression and thus consumes cognitive resources (Gross, 2002; Gross & John, 2003), ultimately leading to increased stress levels and burnout (Chang, 2009, 2013, 2020; Jeon & Ardeleanu, 2020; Lee et al., 2016). Using a daily diary method, Lavy and Eshet (2018) documented the negative spiral of K-12 teachers’ negative emotions and their use of suppression.

In contrast, the use of reappraisal diminishes the negative influence of reencountered emotional information (Blechert et al., 2012; Denny et al., 2015). For instance, it has been shown that reappraisal-related behavioral (negative affect) and neural (right amygdala) effects can last for periods of up to a week, and these enduring neural changes do not require ongoing recruitment of cognitive resources (Denny et al., 2015).

### STEM Teaching-Related Outcomes (Bottom Three Boxes)

Answering calls to incorporate an interpersonal perspective on emotion regulation, which involves regulation processes by and with the help of others (Zaki & Williams, 2013), researchers have recently drawn attention to the effects of teachers’ emotion regulation skills on both themselves and their students (Jeon & Ardeleanu, 2020; Taxer & Gross, 2018). Interpersonal emotion regulation may be particularly beneficial in the STEM classroom. Teachers can be immensely helpful in managing the emotions of learners exposed to sensitive and emotionally-loaded STEM content (Daches Cohen et al., 2021; Hart & Ganley, 2019; Layzer Yavin et al., 2022).

#### Teachers’ Motivation and Well-Being (Bottom Left Box)

Teachers’ emotions have been associated with self-efficacy (Klassen & Chiu, 2010) and self-motivation (Kazén et al., 2015; Parr et al., 2021) and have been shown to affect their professional engagement, burnout, and turnover (Bodenheimer & Shuster, 2020; Buettner et al., 2016; Burić et al., 2019; Taxer & Frenzel, 2015; Torres, 2020). In addition, teachers seem to be more prone to burnout when they frequently regulate emotions by using avoidance or suppression (Carson, 2007; Chang, 2013; Lee et al., 2016; Tsouloupas et al., 2010). Suppression has been linked with increased anxiety (Lee et al., 2016), strain, and emotional exhaustion among teachers (Chang, 2013; Moè & Katz, 2020; Taxer & Frenzel, 2015). Similarly, research on emotional labor in teachers found surface acting, which does not distinguish between faked, hidden, or masked emotions, was negatively associated with teachers’ well-being (Hülsheger et al., 2010; Kenworthy et al., 2014). Hiding, masking, or faking emotional expression can lead to an ongoing internal state of emotional dissonance between the experienced emotion and the outwardly displayed one (Keller et al., 2014). This emotional dissonance can be detrimental to teachers’ occupational well-being (Näring et al., 2006).

Secondary school teachers who use reappraisal to reinterpret stressful classroom situations have been found to demonstrate more positive emotions (Jiang et al., 2016), such as enjoyment (Lee et al., 2016), and manifest less emotional exhaustion (Donker et al., 2020), blunted physiological indicators of chronic stress (Katz et al., 2018), less negative affective experiences in the context of student misbehavior, and less suppression of their in-the-moment negative emotions (Chang & Taxer 2021). In general, teachers at various teaching levels with higher emotion regulation skills report a lower level of depressive and anxious symptoms (Mérida-López et al., 2017).

#### Teachers’ Behaviors (Bottom Middle Box)

The emotional state of teachers is arguably one of the key factors in creating a positive classroom environment (Yan et al., 2011). For example, preschool (Zinsser et al., 2018) and special education (Wong et al., 2017) teachers’ stress has been associated with classroom quality, teachers’ caregiving behaviors, and their relationships with students (Whitaker et al., 2015). When teachers regulate their negative emotions through reappraisal, teacher-student interactions may be more positive (Braun et al., 2019) and teachers may react in a more supportive way to students’ emotions (Jeon et al., 2016; Swartz & McElwain, 2012). Primary school STEM teachers who reported higher enjoyment during teaching sustained their positive attitudes when students struggled, and they spent more time on teaching (Russo et al., 2020), planning instruction, and evaluating teaching goals and teaching strategies supporting self-regulation (Chatzistamatiou et al., 2014).

By implementing reappraisal, teachers may demonstrate more effective pedagogical behaviors (i.e., autonomy supportive and structuring motivating styles; Lee et al., 2016; Moè & Katz, 2020) and greater classroom management efficacy (Sutton & Harper, 2009). Not surprisingly, teachers believe emotion regulation promotes more effective teaching and conforms to their image of an ideal teacher (Sutton, 2004; Sutton & Wheatley, 2003), possibly because of the effects of emotion regulation on the learning atmosphere (Yan et al., 2011). Taxer and Gross (2018) found elementary and secondary school teachers often described their own or their students’ negative emotions as impeding teaching quality and students’ learning, whereas positive emotions fostered teaching quality and student learning.

#### Students (Bottom Right Box)

A teacher’s emotional state can have a central role in students’ academic (Pekrun et al., 2009) and epistemic emotions (Arango-Muñoz, 2014) related to the emotional reaction of students during achievement situations and knowledge acquisition, respectively (Muis et al., 2018). Three aspects of this relationship may be distinguished.

First, teachers’ emotions affect students’ academic and epistemic emotions and perceptions (Becker et al., 2014; Frenzel et al., 2018; Keller & Becker, 2021; Muis et al., 2018; Rodrigo-Ruiz, 2016; Zinsser et al., 2013), a process often referred to as emotion transmission (Frenzel et al., 2018). Both teachers (Taxer & Gross, 2018) and students seem to be aware of the contagious effect of teachers’ emotions (Frenzel et al., 2009; 2018; Oberle et al., 2020). For example, elementary and secondary school teachers’ enjoyment was found to be positively related to students’ perceptions of teachers’ enthusiasm and enjoyment, which, in turn, was positively related to students’ enjoyment (Frenzel et al., 2018; Keller & Becker, 2021). Elementary students of teachers with poor occupational well-being were found to exhibit higher levels of morning cortisol, a biological indicator of stress (Oberle & Schonert-Reichl, 2016). A qualitative study demonstrated that secondary school teachers whose students perceived them as often experiencing negative emotions were hiding, masking, or faking their emotional expression (Jiang et al., 2016). In general, poor occupational well-being among teachers has been related to emotion dysregulation (Chang, 2013; Moè & Katz, 2020; Taxer & Frenzel, 2015). Students’ perceptions of teachers’ emotional dissonance can have negative effects on students’ emotions (Keller & Becker, 2021). In contrast, the degree to which teachers attempt to modify feelings to regulate their emotion (i.e., reappraisal; see Figure 1) has been negatively associated with students’ emotional distress (Braun et al., 2019).

Second, positive emotions in the classroom are at the core of students’ motivation, learning, and academic performance (Renninger & Hidi, 2015; Schubert et al., 2023). Epistemic emotions, such as surprise, confusion, and curiosity, are considered to have functional importance in STEM learning (Schubert et al., 2023; Schukajlow et al., 2017), and it appears that the teacher-student relationship may be a mediating factor (Harding et al., 2019). For example, enjoyment and happiness among preservice STEM teachers significantly improved their behavioral and cognitive engagement in STEM education (Kim et al., 2015). Teachers’ well-being (Malmberg & Hagger, 2009) and teacher-student relationships (e.g., Martin & Collie, 2019; Spilt et al., 2012), in turn, have been significantly associated with students’ long-term growth in academic achievement. Thus, social-emotional aspects of the teacher-student relationship are inherent in many instruction models (Kunter et al., 2013).

Third, teachers dealing with more disruptive classroom behaviors were found to experience poor occupational health and high levels of burnout (Herman et al., 2018; Rodrigo-Ruiz, 2016; Zinsser et al., 2013). Those teachers who also tend to hide, mask, or fake their emotional expression (Chang, 2013; Moè & Katz, 2020; Taxer & Frenzel, 2015) may transfer negative feelings and thoughts to their teaching and struggle with guiding and encouraging students’ expressiveness in challenging situations (Jeon et al., 2016). For example, students have to learn how to regulate and resolve confusion as it arises, as confusion is an unavoidable consequence of learning (Muis et al., 2018). In addition, based on the framework of interpersonal emotion regulation (Zaki & Williams, 2013), by modeling the use of maladaptive forms of emotion regulation, teachers may affect their students’ emotion regulation tendencies (Brady et al., 2018; Fried, 2011; Harley et al., 2019), building inaccurate inferences that do not support social-emotional learning (Braun et al., 2020; Jeon et al., 2019) and engagement (Burić & Frenzel, 2021; Kwon et al., 2017; Sutton & Harper, 2009) and increase emotional distress (Braun et al., 2020).

### Reciprocal Relations between STEM Teachers’ Emotions and Emotion Regulation and STEM Teaching-Related Outcomes (Arrows)

Previous studies have suggested a bidirectional emotion transmission between STEM teachers’ emotions and teacher-related, teaching-related, and student-related outcomes. For example, teachers’ emotions and behaviors, teacher-student interactions (Frenzel et al., 2009, 2018, 2021), and students’ motivation and achievement (Chen, 2019; Uzuntiryaki-Kondakci et al., 2022) may influence the emerging emotions in teachers, with impacts on teachers’ self-concepts (O’Connor, 2008). Teachers’ self-concepts, in turn, may intensify the teachers’ positive emotions (Tsang & Jiang, 2018).

Accordingly, our model suggests that under conditions of stress, a negative cycle can be created: high STEM teachers’ stress levels → disrupted STEM teachers’ emotion regulation resources → STEM teachers’ emotion dysregulation → negative STEM teacher-related, teaching-related, and student-related outcomes → high STEM teachers’ stress levels, and so on.

In sum, emotion regulation (versus dysregulation), particularly reappraisal, is an important skill not only for STEM teachers (Jeon & Ardeleanu, 2020; Taxer & Gross, 2018), but also for their students (e.g., Braun et al., 2019; Oberle & Schonert-Reichl, 2016) and learning processes (e.g., Wong et al., 2017; Zinsser et al., 2018). Despite the environmental stressors in STEM teaching (Schleicher, 2019) and although STEM teachers may have negative feelings (Cowan et al., 2016; Cui et al., 2018; Hembree, 1990) that impair their ability to regulate emotion (e.g., Dixon-Gordon et al., 2015; Levy-Gigi et al., 2016; Shafir et al., 2015, 2016), STEM educators do not generally receive support to develop their own emotional skills (Patti et al., 2015) or those of their students (Reinke et al., 2011). The research on how teachers regulate their emotions and which strategies are effective in the classroom is sparse (Jeon & Ardeleanu, 2020; Taxer & Gross, 2018).

## Discussion

### Implications

To avoid the negative outcomes of teaching-related stress, it is necessary to improve the emotional trajectory at each step in our framework. Actions must be taken to reduce STEM teachers’ job stress or at least to protect against escalating stress. A recent study (Han & Hur, 2021) examining the reasons for STEM teachers’ transition to external industries suggested education policies need to provide more support in areas of career advancement and the creation of autonomous classroom environments. The degree of autonomy in the classroom may be affected by the frequent changes and reforms in STEM education (Li et al., 2020; Stehle & Peters-Burton, 2019), possibly leading to negative emotions among STEM teachers (Jiang et al., 2021; Lee et al., 2013; Tsang & Kwong, 2017). In a qualitative study (Fisher & Royster, 2016), secondary math teachers proposed ways to alleviate their stress, including more support with student discipline problems, fewer events and meetings after school hours, and less paperwork and extra duties. Future studies in this direction may shed further light on ways to reduce STEM teachers’ work-related stress.

Previous work on reducing teachers’ occupational stress has included organization-level (Naghieh et al., 2015) and person-level approaches (Iancu et al., 2018). The limitations of such interventions include the need for extensive financial and organizational resources (Awa et al., 2010) and small levels of efficiency (Iancu et al., 2018). Person-level approaches include knowledge-based (i.e., informational or psychosocial training; Cicotto et al., 2014), behavioral (i.e., techniques to reduce stress; Chan, 2011), cognitive-behavioral (i.e., cognitive training and practice in behavioral strategies; Ebert et al., 2014), and mindfulness-based interventions (i.e., focusing on the process of feeling and thinking; Beshai et al., 2016).

Although emotion regulation skills have far-reaching implications in the context of teaching and learning (Braun et al., 2019; Frenzel et al., 2021; Jeon et al., 2016; Jiang et al., 2016; Lee et al., 2016; Moè & Katz, 2020; Sutton & Harper, 2009), and teachers consider these skills to be extremely important (Sutton, 2004; Sutton & Wheatley, 2003), there is a lack of such interventions for teachers (Chen & Cheng, 2022; Patti et al., 2015; Reinke et al., 2011; Uitto et al., 2015). As mentioned above, evidence suggests suppression is the most frequently used emotion regulation strategy in classroom situations (Gong et al., 2013; Keller et al., 2014; Taxer & Frenzel, 2015; Taxer & Gross, 2018), even though teachers have a wide repertoire of emotion regulation strategies (Chang & Taxer, 2021; Taxer & Gross, 2018). Thus, teachers could benefit from understanding how to use reappraisal in different situations in the classroom context (Taxer & Gross, 2018). Specifically, they should be familiar with the ways to use reappraisal in response to student misbehavior, an emotion-evoking situation that frequently causes teachers to use suppression (Jeon & Ardeleanu, 2020; Jeon et al., 2016; Taxer & Gross, 2018).

Reappraisal-based interventions for STEM teachers may benefit from the existing studies on similar interventions among students. Such interventions have multiple advantages. First, managing emotions is an integral part of teachers’ work (Lee et al., 2016). Second, reappraisal-focused interventions have translatability from laboratory to field contexts (Jamieson et al., 2016). Third, the interventions are generally short-term, non-invasive, simple to implement, and require limited time and resources from participants, making them perfectly tailored to the educational setting. Fourth, the work is grounded on strong theory (Jamieson et al., 2010). Fifth, the interventions promote leading principles of the educational system: innovation and relevance in education and learning, autonomy and trust of teachers, as well as equal opportunities for children to acquire valuable skills even when they involve engagement in stressful pursuits.

Note that such interventions may need to be adapted in different cultures because cultural norms shape individuals’ perceptions about the appropriate expression of emotions (i.e., emotional display rules) and which emotion regulation strategies are adaptive or maladaptive (Butler et al., 2007; Ford & Gross, 2019; Ford & Mauss, 2015; Hagenauer et al., 2016; Schutz et al., 2009). In addition, regulating negative emotions is not necessarily adaptive; these emotions sometimes provide important information about the best response in a given situation (Feinberg et al., 2020; Ford & Troy 2019). Therefore, an effective therapeutic approach might emphasize the functionality of negative emotions and encourage an appreciation of their usefulness, alongside promoting a reappraisal of negative emotions that create a negative bias in thoughts and behavior (Feinberg et al., 2020).

Considerable evidence in the literature confirms the validity of the units in our STEM-MENTOR model indicating the critical role of STEM teachers’ emotion regulation knowledge and abilities. Simply stated, effective emotion regulation yields benefits to teachers and students alike. However, the model should be tested in follow-up studies, especially meta-analyses examining the variables simultaneously in different cultures and at diverse educational levels. Our model may be fertile ground for research and interventions that will promote STEM teachers’ well-being, thus improving students’ epistemological experience and achievements.

## Conclusion

Because of the emotional demands of STEM teaching, STEM teachers face a wide range of stressors, including low student achievement and negative attitudes towards STEM subjects. Applying the process model of emotion regulation to the STEM teaching context, the STEM-MENTOR framework designates how contextual factors increase STEM teachers’ stress and how STEM teaching-related stress impairs emotion regulation resources, thereby promoting emotion dysregulation. Importantly, we emphasize the effects of STEM teachers’ intensified negative emotions and emotion dysregulation not only on themselves, but also on their students’ emotions, behaviors, and learning processes. Given the positive emotional and academic outcomes of stress reappraisal interventions in the STEM fields of study, we suggest that future research should focus on developing STEM teachers’ emotion regulation knowledge and abilities.

## Author Information

LDC is part of the Edmond J. Safra Brain Research Center for the Study of Learning Disabilities. She studies the role of emotion regulation in the development of math anxiety and whether adaptive emotion regulation strategies can be developed in math-anxious individuals. JG is a leader in the areas of emotion and emotion regulation and has published nearly 600 frequently cited papers on emotion regulation and received numerous teaching and research awards. By integrating behavioural, physiological, and brain measures, his research seeks to clarify emotion-related personality processes and individual differences. OR is part of the Edmond J. Safra Brain Research Center for the Study of Learning Disabilities. She is a leading figure in the areas of typical and atypical development of numerical cognition, learning disabilities (e.g., developmental dyscalculia and ADHD) and math anxiety.

## References

[1] Akiba, M., Chiu, Y. L., Shimizu, K., & Liang, G. (2012). Teacher salary and national achievement: A cross-national analysis of 30 countries. International Journal of Educational Research, 53, 171–181. DOI: 10.1016/j.ijer.2012.03.007

[2] Akinsola, M. K. (2008). Relationship of some psychological variables in predicting problem solving ability of in-service mathematics teachers. The Mathematics Enthusiast, 5(1), 79–100. DOI: 10.54870/1551-3440.1088

[3] Aldao, A., & Nolen-Hoeksema, S. (2013). One versus many: Capturing the use of multiple emotion regulation strategies in response to an emotion-eliciting stimulus. Cognition & Emotion, 27(4), 753–760. DOI: 10.1080/02699931.2012.739998 23130665

[4] Aldao, A., Sheppes, G., & Gross, J. J. (2015). Emotion regulation flexibility. Cognitive Therapy and Research, 39(3), 263–278. DOI: 10.1007/s10608-014-9662-4

[5] Aldrup, K., Klusmann, U., Lüdtke, O., Göllner, R., & Trautwein, U. (2018). Student misbehavior and teacher well-being: Testing the mediating role of the teacher-student relationship. Learning and Instruction, 58, 126–136. DOI: 10.1016/j.learninstruc.2018.05.006

[6] American Federation of Teachers. (2017). 2017 educator quality of work life survey. American Federation of Teachers.

[7] Arango-Muñoz, S. (2014). The nature of epistemic feelings. Philosophical Psychology, 27(2), 193–211. DOI: 10.1080/09515089.2012.732002

[8] Awa, W. L., Plaumann, M., & Walter, U. (2010). Burnout prevention: A review of intervention programs. Patient Education and Counseling, 78(2), 184–190. DOI: 10.1016/j.pec.2009.04.008 19467822

[9] Balzarotti, S., Chiarella, V., & Ciceri, M. R. (2017). Individual differences in cognitive reappraisal predict emotional experience prior to achievement situations. Journal of Individual Differences, 38(3), 144–154. DOI: 10.1027/1614-0001/a000231

[10] Banich, M. T., Mackiewicz, K. L., Depue, B. E., Whitmer, A. J., Miller, G. A., & Heller, W. (2009). Cognitive control mechanisms, emotion and memory: A neural perspective with implications for psychopathology. Neuroscience & Biobehavioral Reviews, 33(5), 613–630. DOI: 10.1016/j.neubiorev.2008.09.010 18948135 PMC2865433

[11] Barroso, C., Ganley, C. M., McGraw, A. L., Geer, E. A., Hart, S. A., & Daucourt, M. C. (2021). A meta-analysis of the relation between math anxiety and math achievement. Psychological Bulletin, 147(2), 134–168. DOI: 10.1037/bul0000307 33119346 PMC8300863

[12] Becker, E. S., Goetz, T., Morger, V., & Ranellucci, J. (2014). The importance of teachers’ emotions and instructional behavior for their students’ emotions–An experience sampling analysis. Teaching and Teacher Education, 43, 15–26. DOI: 10.1016/j.tate.2014.05.002

[13] Beilock, S. L., Gunderson, E. A., Ramirez, G., & Levine, S. C. (2010). Female teachers’ math anxiety affects girls’ math achievement. Proceedings of the National Academy of Sciences, 107(5), 1860–1863. DOI: 10.1073/pnas.0910967107 PMC2836676 20133834

[14] Beilock, S. L., & Willingham, D. T. (2014). Math anxiety: Can teachers help students reduce it? Ask the cognitive scientist. American Educator, 38(2), 28–32.

[15] Bellocchi, A., Quigley, C. F., & Otrel-Cass, K. (2017). Emotions, aesthetics and wellbeing in science education: Theoretical foundations. In A. Bellocchi, C. Quigley, & K. Otrel-Cass (Eds.), Exploring emotions, aesthetics and wellbeing in science education research (pp. 1–9). Springer. DOI: 10.1007/978-3-319-43353-0_1

[16] Beshai, S., McAlpine, L., Weare, K., & Kuyken, W. (2016). A non-randomised feasibility trial assessing the efficacy of a mindfulness-based intervention for teachers to reduce stress and improve well-being. Mindfulness, 7(1), 198–208. DOI: 10.1007/s12671-015-0436-1

[17] Bigman, Y. E., Sheppes, G., & Tamir, M. (2017). When less is more: Effects of the availability of strategic options on regulating negative emotions. Emotion, 17(6), 993–1006. DOI: 10.1037/emo0000303 28277712

[18] Blechert, J., Sheppes, G., Di Tella, C., Williams, H., & Gross, J. J. (2012). See what you think: Reappraisal modulates behavioral and neural responses to social stimuli. Psychological Science, 23(4), 346–353. DOI: 10.1177/0956797612438559 22431908

[19] Bodenheimer, G., & Shuster, S. M. (2020). Emotional labour, teaching and burnout: Investigating complex relationships. Educational Research, 62(1), 63–76. DOI: 10.1080/00131881.2019.1705868

[20] Bonanno, G. A., & Burton, C. L. (2013). Regulatory flexibility: An individual differences perspective on coping and emotion regulation. Perspectives on Psychological Science, 8(6), 591–612. DOI: 10.1177/1745691613504116 26173226

[21] Bonanno, G. A., Romero, S. A., & Klein, S. I. (2015). The temporal elements of psychological resilience: An integrative framework for the study of individuals, families, and communities. Psychological Inquiry, 26(2), 139–169. DOI: 10.1080/1047840X.2015.992677

[22] Bradley, M. M., Miccoli, L., Escrig, M. A., & Lang, P. J. (2008). The pupil as a measure of emotional arousal and autonomic activation. Psychophysiology, 45(4), 602–607. DOI: 10.1111/j.1469-8986.2008.00654.x 18282202 PMC3612940

[23] Brady, S. T., Hard, B. M., & Gross, J. J. (2018). Reappraising test anxiety increases academic performance of first-year college students. Journal of Educational Psychology, 110(3), 395–406. DOI: 10.1037/edu0000219

[24] Brandenburg, R., McDonough, S., Burke, J., & White, S. (2016). Teacher education: Innovation, intervention and impact. Springer. DOI: 10.1007/978-981-10-0785-9

[25] Braun, S. S., Roeser, R. W., Mashburn, A. J., & Skinner, E. (2019). Middle school teachers’ mindfulness, occupational health and well-being, and the quality of teacher-student interactions. Mindfulness, 10(2), 245–255. DOI: 10.1007/s12671-018-0968-2

[26] Braun, S. S., Schonert-Reichl, K. A., & Roeser, R. W. (2020). Effects of teachers’ emotion regulation, burnout, and life satisfaction on student well-being. Journal of Applied Developmental Psychology, 69, 101151. DOI: 10.1016/j.appdev.2020.101151

[27] Brooks, A. W. (2014). Get excited: Reappraising pre-performance anxiety as excitement. Journal of Experimental Psychology: General, 143(3), 1144–1158. DOI: 10.1037/a0035325 24364682

[28] Buettner, C. K., Jeon, L., Hur, E., & Garcia, R. E. (2016). Teachers’ social–emotional capacity: Factors associated with teachers’ responsiveness and professional commitment. Early Education and Development, 27(7), 1018–1039. DOI: 10.1080/10409289.2016.1168227

[29] Buhle, J. T., Silvers, J. A., Wager, T. D., Lopez, R., Onyemekwu, C., Kober, H., Weber, J., & Ochsner, K. N. (2014). Cognitive reappraisal of emotion: A meta-analysis of human neuroimaging studies. Cerebral Cortex, 24(11), 2981–2990. DOI: 10.1093/cercor/bht154 23765157 PMC4193464

[30] Burić, I. (2019). The role of emotional labor in explaining teachers’ enthusiasm and students’ outcomes: A multilevel mediational analysis. Learning and Individual Differences, 70, 12–20. DOI: 10.1016/j.lindif.2019.01.002

[31] Burić, I., & Frenzel, A. C. (2021). Teacher emotional labour, instructional strategies, and students’ academic engagement: A multilevel analysis. Teachers and Teaching, 27(5), 335–352. DOI: 10.1080/13540602.2020.1740194

[32] Burić, I., Kim, L. E., & Hodis, F. (2021). Emotional labor profiles among teachers: Associations with positive affective, motivational, and well-being factors. Journal of Educational Psychology, 113(6), 1227–1243. DOI: 10.1037/edu0000654

[33] Burić, I., Slišković, A., & Penezić, Z. (2019). Understanding teacher well-being: A cross-lagged analysis of burnout, negative student-related emotions, psychopathological symptoms, and resilience. Educational Psychology, 39(9), 1136–1155. DOI: 10.1080/01443410.2019.1577952

[34] Bursal, M., & Paznokas, L. (2006). Mathematics anxiety and preservice elementary teachers’ confidence to teach mathematics and science. School Science and Mathematics, 106(4), 173–180. DOI: 10.1111/j.1949-8594.2006.tb18073.x

[35] Butler, E. A., Lee, T. L., & Gross, J. J. (2007). Emotion regulation and culture: Are the social consequences of emotion suppression culture-specific? Emotion, 7(1), 30–48. DOI: 10.1037/1528-3542.7.1.30 17352561

[36] Butler, R. (2012). Striving to connect: Extending an achievement goal approach to teacher motivation to include relational goals for teaching. Journal of Educational Psychology, 104(3), 726–742. DOI: 10.1037/a0028613

[37] Bybee, R. W. (2013). The case for STEM education: Challenges and opportunities. NSTA Press.

[38] Carson, R. L. (2007). Emotional regulation and teacher burnout: Who says that the management of emotional expression doesn’t matter? Paper presented at the American Educational Research Association Annual Meeting. Chicago, IL.

[39] Chan, D. W. (2006). Emotional intelligence and components of burnout among Chinese secondary school teachers in Hong Kong. Teaching and Teacher Education, 22(8), 1042–1054. DOI: 10.1016/j.tate.2006.04.005

[40] Chan, D. W. (2011). Burnout and life satisfaction: Does gratitude intervention make a difference among Chinese school teachers in Hong Kong? Educational Psychology, 31(7), 809–823. DOI: 10.1080/01443410.2011.608525

[41] Chang, M. L. (2009). An appraisal perspective of teacher burnout: Examining the emotional work of teachers. Educational Psychology Review, 21(3), 193–218. DOI: 10.1007/s10648-009-9106-y

[42] Chang, M. L. (2013). Toward a theoretical model to understand teacher emotions and teacher burnout in the context of student misbehavior: Appraisal, regulation and coping. Motivation and Emotion, 37(4), 799–817. DOI: 10.1007/s11031-012-9335-0

[43] Chang, M. L. (2020). Emotion display rules, emotion regulation, and teacher burnout. Frontiers in Education, 5, 90. DOI: 10.3389/feduc.2020.00090

[44] Chang, M. L., & Taxer, J. (2021). Teacher emotion regulation strategies in response to classroom misbehavior. Teachers and Teaching, 27(5), 353–369. DOI: 10.1080/13540602.2020.1740198

[45] Chatzistamatiou, M., Dermitzaki, I., & Bagiatis, V. (2014). Self-regulatory teaching in mathematics: Relations to teachers’ motivation, affect and professional commitment. European Journal of Psychology of Education, 29(2), 295–310. DOI: 10.1007/s10212-013-0199-9

[46] Chen, J. (2019). Exploring the impact of teacher emotions on their approaches to teaching: A structural equation modelling approach. British Journal of Educational Psychology, 89(1), 57–74. DOI: 10.1111/bjep.12220 29603123

[47] Chen, J., & Cheng, T. (2022). Review of research on teacher emotion during 1985–2019: A descriptive quantitative analysis of knowledge production trends. European Journal of Psychology of Education, 37(2), 417–438. DOI: 10.1007/s10212-021-00537-1 PMC7957038 40477211

[48] Choe, K. W., Jenifer, J. B., Rozek, C. S., Berman, M. G., & Beilock, S. L. (2019). Calculated avoidance: Math anxiety predicts math avoidance in effort-based decision-making. Science Advances, 5(11), eaay1062. DOI: 10.1126/sciadv.aay1062 31799398 PMC6867883

[49] Cicotto, G., De Simone, S., Giustiniano, L., & Pinna, R. (2014). Psychosocial training: A case of self-efficacy improvement in an Italian school. Journal of Change Management, 14(4), 475–499. DOI: 10.1080/14697017.2014.978536

[50] Cohen, N., & Mor, N. (2018). Enhancing reappraisal by linking cognitive control and emotion. Clinical Psychological Science, 6(1), 155–163. DOI: 10.1177/2167702617731379

[51] Cohen, N., Mor, N., & Henik, A. (2015). Linking executive control and emotional response: A training procedure to reduce rumination. Clinical Psychological Science, 3(1), 15–25. DOI: 10.1177/2167702614530114

[52] Collie, R. J., & Mansfield, C. F. (2022). Teacher and school stress profiles: A multilevel examination and associations with work-related outcomes. Teaching and Teacher Education, 116, 103759. DOI: 10.1016/j.tate.2022.103759

[53] Collie, R. J., Shapka, J. D., & Perry, N. E. (2012). School climate and social–emotional learning: Predicting teacher stress, job satisfaction, and teaching efficacy. Journal of Educational Psychology, 104(4), 1189–1204. DOI: 10.1037/a0029356

[54] Conte, B., Hahnel, U. J., & Brosch, T. (2022). From values to emotions: Cognitive appraisal mediates the impact of core values on emotional experience. Emotion. Advance online publication. DOI: 10.1037/emo0001083 35389734

[55] Cowan, J., Goldhaber, D., Hayes, K., & Theobald, R. (2016). Missing elements in the discussion of teacher shortages. Educational Researcher, 45(8), 460–462. DOI: 10.3102/0013189X16679145

[56] Cui, Q., Chao, Q., Han, J., Zhang, X., Ren, Y., & Shi, J. (2018). Job stress, burnout and the relationship among the science and mathematics teachers in basic education schools. EURASIA Journal of Mathematics, Science and Technology Education, 14(7), 3235–3244. DOI: 10.29333/ejmste/85957

[57] Daches, S., & Mor, N. (2014). Training ruminators to inhibit negative information: A preliminary report. Cognitive Therapy and Research, 38(2), 160–171. DOI: 10.1007/s10608-013-9585-5

[58] Daches Cohen, L., Layzer Yavin, L., & Rubinsten, O. (2021). Females’ negative affective valence to math-related words. Acta Psychologica, 217, 103313. DOI: 10.1016/j.actpsy.2021.103313 33930625

[59] Daches Cohen, L., & Rubinsten, O. (2017). Mothers, intrinsic math motivation, arithmetic skills, and math anxiety in elementary school. Frontiers in Psychology, 8, 1939. DOI: 10.3389/fpsyg.2017.01939 29180973 PMC5693896

[60] Daches Cohen, L., & Rubinsten, O. (2022). Math anxiety and deficient executive control: Does reappraisal modulate this link? Annals of the New York Academy of Sciences. Epub ahead of print. PMID: 35389529. DOI: 10.1111/nyas.14772 PMC9544869 35389529

[61] De Lissnyder, E., Koster, E. H., Goubert, L., Onraedt, T., Vanderhasselt, M. A., & De Raedt, R. (2012). Cognitive control moderates the association between stress and rumination. Journal of Behavior Therapy and Experimental Psychiatry, 43(1), 519–525. DOI: 10.1016/j.jbtep.2011.07.004 21813083

[62] Deligkaris, P., Panagopoulou, E., Montgomery, A. J., & Masoura, E. (2014). Job burnout and cognitive functioning: A systematic review. Work & Stress, 28(2), 107–123.

[63] Denham, S. A., Bassett, H. H., & Zinsser, K. (2012). Early childhood teachers as socializers of young children’s emotional competence. Early Childhood Education Journal, 40(3), 137–143. DOI: 10.1007/s10643-012-0504-2

[64] Denny, B. T., Inhoff, M. C., Zerubavel, N., Davachi, L., & Ochsner, K. N. (2015). Getting over it: Long-lasting effects of emotion regulation on amygdala response. Psychological Science, 26(9), 1377–1388. DOI: 10.1177/0956797615578863 26231911 PMC4567486

[65] Devine, A., Hill, F., Carey, E., & Szűcs, D. (2018). Cognitive and emotional math problems largely dissociate: Prevalence of developmental dyscalculia and mathematics anxiety. Journal of Educational Psychology, 110(3), 431–444. DOI: 10.1037/edu0000222

[66] Dixon-Gordon, K. L., Aldao, A., & De Los Reyes, A. (2015). Emotion regulation in context: Examining the spontaneous use of strategies across emotional intensity and type of emotion. Personality and Individual Differences, 86, 271–276. DOI: 10.1016/j.paid.2015.06.011

[67] Donker, M. H., Erisman, M. C., Van Gog, T., & Mainhard, T. (2020). Teachers’ emotional exhaustion: Associations with their typical use of and implicit attitudes toward emotion regulation strategies. Frontiers in Psychology, 11, 867. DOI: 10.3389/fpsyg.2020.00867 32547437 PMC7273523

[68] Dorman Ilan, S., Tamuz, N., & Sheppes, G. (2019). The fit between emotion regulation choice and individual resources is associated with adaptive functioning among young children. Cognition and Emotion, 33(3), 597–605. DOI: 10.1080/02699931.2018.1470494 29733245

[69] Ebert, D. D., Lehr, D., Boß, L., Riper, H., Cuijpers, P., Andersson, G., Thiart, H., Heber, E., & & Berking, M. (2014). Efficacy of an internet-based problem-solving training for teachers: Results of a randomized controlled trial. Scandinavian Journal of Work, Environment & Health, 582–596. DOI: 10.5271/sjweh.3449 25121986

[70] Epel, E. S., Crosswell, A. D., Mayer, S. E., Prather, A. A., Slavich, G. M., Puterman, E., & Mendes, W. B. (2018). More than a feeling: A unified view of stress measurement for population science. Frontiers in Neuroendocrinology, 49, 146–169. DOI: 10.1016/j.yfrne.2018.03.001 29551356 PMC6345505

[71] Escalante Mateos, N., Fernández-Zabala, A., & Goñi Palacios, E. (2021). School climate and perceived academic performance: Direct or resilience-mediated relationship? Sustainability, 13(1), 68. DOI: 10.3390/su13010068

[72] Estapa, A. T., & Tank, K. M. (2017). Supporting integrated STEM in the elementary classroom: a professional development approach centered on an engineering design challenge. International Journal of STEM education, 4(1), 1–16. DOI: 10.1186/s40594-017-0058-3

[73] Feinberg, M., Ford, B. Q., & Flynn, F. J. (2020). Rethinking reappraisal: The double-edged sword of regulating negative emotions in the workplace. Organizational Behavior and Human Decision Processes, 161, 1–19. DOI: 10.1016/j.obhdp.2020.03.005

[74] Feldman, J. L., & Freitas, A. L. (2021). The generality of effects of emotional experience on emotion-regulation choice. Emotion, 21(1), 211–219. DOI: 10.1037/emo0000611 31192667

[75] Fisher, M. H., & Royster, D. (2016). Mathematics teachers’ support and retention: Using Maslow’s hierarchy to understand teachers’ needs. International Journal of Mathematical Education in Science and Technology, 47(7), 993–1008. DOI: 10.1080/0020739X.2016.1162333

[76] Foley, A. E., Herts, J. B., Borgonovi, F., Guerriero, S., Levine, S. C., & Beilock, S. L. (2017). The math anxiety-performance link: A global phenomenon. Current Directions in Psychological Science, 26(1), 52–58. DOI: 10.1177/0963721416672463

[77] Ford, B. Q., & Gross, J. J. (2019). Why beliefs about emotion matter: An emotion-regulation perspective. Current Directions in Psychological Science, 28(1), 74–81. DOI: 10.1177/0963721418806697

[78] Ford, B. Q., Gross, J. J., & Gruber, J. (2019). Broadening our field of view: The role of emotion polyregulation. Emotion Review, 11(3), 197–208. DOI: 10.1177/1754073919850314

[79] Ford, B. Q., & Mauss, I. B. (2015). Culture and emotion regulation. Current Opinion in Psychology, 3, 1–5. DOI: 10.1016/j.copsyc.2014.12.004 25729757 PMC4341898

[80] Ford, B. Q., & Troy, A. S. (2019). Reappraisal reconsidered: A closer look at the costs of an acclaimed emotion-regulation strategy. Current Directions in Psychological Science, 28(2), 195–203. DOI: 10.1177/0963721419827526

[81] Frenzel, A. C., Becker-Kurz, B., Pekrun, R., Goetz, T., & Lüdtke, O. (2018). Emotion transmission in the classroom revisited: a reciprocal effects model of teacher and student enjoyment. Journal of Educational Psychology, 110(5), 628–639. DOI: 10.1037/edu0000228

[82] Frenzel, A. C., Daniels, L., & Burić, I. (2021). Teacher emotions in the classroom and their implications for students. Educational Psychologist, 56(4), 250–264. DOI: 10.1080/00461520.2021.1985501

[83] Frenzel, A. C., Goetz, T., Lüdtke, O., Pekrun, R., & Sutton, R. E. (2009). Emotional transmission in the classroom: Exploring the relationship between teacher and student enjoyment. Journal of Educational Psychology, 101(3), 705–716. DOI: 10.1037/a0014695

[84] Frenzel, A. C., Pekrun, R., Goetz, T., Daniels, L. M., Durksen, T. L., Becker-Kurz, B., & Klassen, R. M. (2016). Measuring teachers’ enjoyment, anger, and anxiety: The Teacher Emotions Scales (TES). Contemporary Educational Psychology, 46, 148–163. DOI: 10.1016/j.cedpsych.2016.05.003

[85] Fried, L. (2011). Teaching teachers about emotion regulation in the classroom. Australian Journal of Teacher Education, 36(3), 117–127. DOI: 10.14221/ajte.2011v36n3.1

[86] Friedman-Krauss, A. H., Raver, C. C., Neuspiel, J. M., & Kinsel, J. (2014). Child behavior problems, teacher executive functions, and teacher stress in Head Start classrooms. Early Education and Development, 25(5), 681–702. DOI: 10.1080/10409289.2013.825190 28596698 PMC5460986

[87] Fung, D., Kutnick, P., Mok, I., Leung, F., Lee, B. P. Y., Mai, Y. Y., & Tyler, M. T. (2017). Relationships between teachers’ background, their subject knowledge and pedagogic efficacy, and pupil achievement in primary school mathematics in Hong Kong: An indicative study. International Journal of Educational Research, 81, 119–130. DOI: 10.1016/j.ijer.2016.11.003

[88] Gallup. (2014). State of America’s schools: The path to winning again in education. Gallup, Inc.

[89] Ganley, C. M., Conlon, R. A., McGraw, A. L., Barroso, C., & Geer, E. A. (2021). The effect of brief anxiety interventions on reported anxiety and math test performance. Journal of Numerical Cognition, 7(1), 4–19. DOI: 10.5964/jnc.6065

[90] Ganley, C. M., Schoen, R. C., LaVenia, M., & Tazaz, A. M. (2019). The construct validation of the math anxiety scale for teachers. Aera Open, 5(1), 2332858419839702. DOI: 10.1177/2332858419839702

[91] Gardner, H. (1995). Reflections on multiple intelligences. Phi Delta Kappan, 77(3), 200–208.

[92] Gastaldi, F. G. M., Pasta, T., Longobardi, C., Prino, L. E., & Quaglia, R. (2014). Measuring the influence of stress and burnout in teacher-child relationship. European Journal of Education and Psychology, 7(1), 17–28.

[93] Goldin, P. R., Moodie, C. A., & Gross, J. J. (2019). Acceptance versus reappraisal: Behavioral, autonomic, and neural effects. Cognitive, Affective, & Behavioral Neuroscience, 19(4), 927–944. DOI: 10.3758/s13415-019-00690-7 30656602

[94] Golkar, A., Johansson, E., Kasahara, M., Osika, W., Perski, A., & Savic, I. (2014). The influence of work-related chronic stress on the regulation of emotion and on functional connectivity in the brain. PloS One, 9(9), e104550. DOI: 10.1371/journal.pone.0104550 25184294 PMC4153588

[95] Gong, S., Chai, X., Duan, T., Zhong, L., & Jiao, Y. (2013). Chinese teachers’ emotion regulation goals and strategies. Psychology, 4(11), 870–877. DOI: 10.4236/psych.2013.411125

[96] Greenaway, K. H., Kalokerinos, E. K., & Williams, L. A. (2018). Context is everything (in emotion research). Social and Personality Psychology Compass, 12(6), e12393. DOI: 10.1111/spc3.12393

[97] Greenberg, M. T., Brown, J. L., & Abenavoli, R. M. (2016). Teacher stress and health effects on teachers, students, and schools. Edna Bennett Pierce Prevention Research Center, Pennsylvania State University.

[98] Gresham, G. (2008). Mathematics anxiety and mathematics teacher efficacy in elementary pre-service teachers. Teaching Education, 19(3), 171–184. DOI: 10.1080/10476210802250133

[99] Grillon, C., Quispe-Escudero, D., Mathur, A., & Ernst, M. (2015). Mental fatigue impairs emotion regulation. Emotion, 15(3), 383–389. DOI: 10.1037/emo0000058 25706833 PMC4437828

[100] Grommisch, G., Koval, P., Hinton, J. D., Gleeson, J., Hollenstein, T., Kuppens, P., & Lischetzke, T. (2020). Modeling individual differences in emotion regulation repertoire in daily life with multilevel latent profile analysis. Emotion, 20(8), 1462–1474. DOI: 10.1037/emo0000669 31478725

[101] Gross, J. J. (2002). Emotion regulation: Affective, cognitive, and social consequences. Psychophysiology, 39(3), 281–291. DOI: 10.1017/S0048577201393198 12212647

[102] Gross, J. J. (2013). Emotion regulation: taking stock and moving forward. Emotion, 13(3), 359–365. DOI: 10.1037/a0032135 23527510

[103] Gross, J. J. (2015). Emotion regulation: Current status and future prospects. Psychological Inquiry, 26(1), 1–26. DOI: 10.1080/1047840X.2014.940781

[104] Gross, J. J. (in press). Emotion regulation: Conceptual foundations. In J. J. Gross & B. Q. Ford (Eds.). Handbook of emotion regulation (3rd edition). New York, NY: Guilford Press.

[105] Gross, J. J., & Jazaieri, H. (2014). Emotion, emotion regulation, and psychopathology: An affective science perspective. Clinical Psychological Science, 2(4), 387–401. DOI: 10.1177/2167702614536164

[106] Gross, J. J., & John, O. P. (2003). Individual differences in two emotion regulation processes: implications for affect, relationships, and well-being. Journal of Personality and Social Psychology, 85(2), 348–362. DOI: 10.1037/0022-3514.85.2.348 12916575

[107] Hafni, R. N., Herman, T., Nurlaelah, E., & Mustikasari, L. (2020). The importance of science, technology, engineering, and mathematics (STEM) education to enhance students’ critical thinking skill in facing the industry 4.0. Journal of Physics: Conference Series, 1521(4), 042040. DOI: 10.1088/1742-6596/1521/4/042040

[108] Hagenauer, G., Gläser-Zikuda, M., & Volet, S. E. (2016). University teachers’ perceptions of appropriate emotion display and high-quality teacher-student relationship: Similarities and differences across cultural-educational contexts. Frontline Learning Research, 4(3), 44–74. DOI: 10.14786/flr.v4i3.236

[109] Hagenauer, G., Hascher, T., & Volet, S. E. (2015). Teacher emotions in the classroom: associations with students’ engagement, classroom discipline and the interpersonal teacher-student relationship. European Journal of Psychology of Education, 30(4), 385–403. DOI: 10.1007/s10212-015-0250-0

[110] Haines, S. J., Gleeson, J., Kuppens, P., Hollenstein, T., Ciarrochi, J., Labuschagne, I., Grace, C., & Koval, P. (2016). The wisdom to know the difference: Strategy-situation fit in emotion regulation in daily life is associated with well-being. Psychological Science, 27(12), 1651–1659. DOI: 10.1177/0956797616669086 27738099

[111] Han, D., & Hur, H. (2021). Managing turnover of STEM teacher workforce. Education and Urban Society, 54(2), 205–222. DOI: 10.1177/00131245211053562

[112] Harding, S., Morris, R., Gunnell, D., Ford, T., Hollingworth, W., Tilling, K., Evans, R., Bell, S., Grey, J., Brockman, R., Campbell, R., Araya, R., Murphy, S., & Kidger, J. (2019). Is teachers’ mental health and wellbeing associated with students’ mental health and wellbeing? Journal of Affective Disorders, 242, 180–187. DOI: 10.1016/j.jad.2018.08.080 30189355

[113] Hargreaves, A. (2000). Mixed emotions: Teachers’ perceptions of their interactions with students. Teaching and Teacher Education, 16(8), 811–826. DOI: 10.1016/S0742-051X(00)00028-7

[114] Harley, J. M., Pekrun, R., Taxer, J. L., & Gross, J. J. (2019). Emotion regulation in achievement situations: An integrated model. Educational Psychologist, 54(2), 106–126. DOI: 10.1080/00461520.2019.1587297

[115] Hart, S. A., & Ganley, C.M. (2019). The nature of math anxiety in adults: Prevalence and correlates. Journal of Numerical Cognition, 5(2), 122–139. DOI: 10.5964/jnc.v5i2.195 33842689 PMC8034611

[116] Hembree, R. (1990). The nature, effects, and relief of mathematics anxiety. Journal for Research in Mathematics Education, 21(1), 33–46. DOI: 10.2307/749455

[117] Herman, K. C., Hickmon-Rosa, J. E., & Reinke, W. M. (2018). Empirically derived profiles of teacher stress, burnout, self-efficacy, and coping and associated student outcomes. Journal of Positive Behavior Interventions, 20(2), 90–100. DOI: 10.1177/1098300717732066

[118] Holmqvist, M. (2019). Lack of qualified teachers: A global challenge for future knowledge development. Teacher Education in the 21st Century, 1–13. DOI: 10.5772/intechopen.83417

[119] Hu, T., Zhang, D., Wang, J., Mistry, R., Ran, G., & Wang, X. (2014). Relation between emotion regulation and mental health: A meta-analysis review. Psychological Reports, 114(2), 341–362. DOI: 10.2466/03.20.PR0.114k22w4 24897894

[120] Hülsheger, U. R., Lang, J. W., & Maier, G. W. (2010). Emotional labor, strain, and performance: Testing reciprocal relationships in a longitudinal panel study. Journal of Occupational Health Psychology, 15(4), 505–521. DOI: 10.1037/a0021003 21058862

[121] Iancu, A. E., Rusu, A., Măroiu, C., Păcurar, R., & Maricuțoiu, L. P. (2018). The effectiveness of interventions aimed at reducing teacher burnout: A meta-analysis. Educational Psychology Review, 30(2), 373–396. DOI: 10.1007/s10648-017-9420-8

[122] Jamieson, J. P., Mendes, W. B., Blackstock, E., & Schmader, T. (2010). Turning the knots in your stomach into bows: Reappraising arousal improves performance on the GRE. Journal of Experimental Social Psychology, 46(1), 208–212. DOI: 10.1016/j.jesp.2009.08.015 20161454 PMC2790291

[123] Jamieson, J. P., Mendes, W. B., & Nock, M. K. (2013). Improving acute stress responses: The power of reappraisal. Current Directions in Psychological Science, 22(1), 51–56. DOI: 10.1177/0963721412461500

[124] Jamieson, J. P., Peters, B. J., Greenwood, E. J., & Altose, A. J. (2016). Reappraising stress arousal improves performance and reduces evaluation anxiety in classroom exam situations. Social Psychological and Personality Science, 7, 579–587. DOI: 10.1177/1948550616644656

[125] Jennings, P. A., Doyle, S., Oh, Y., Rasheed, D., Frank, J. L., & Brown, J. L. (2019). Long-term impacts of the CARE program on teachers’ self-reported social and emotional competence and well-being. Journal of School Psychology, 76, 186–202. DOI: 10.1016/j.jsp.2019.07.009 31759466

[126] Jeon, L., & Ardeleanu, K. (2020). Work climate in early care and education and teachers’ stress: Indirect associations through emotion regulation. Early Education and Development, 31(7), 1031–1051. DOI: 10.1080/10409289.2020.1776809

[127] Jeon, L., Buettner, C. K., Grant, A. A., & Lang, S. N. (2019). Early childhood teachers’ stress and children’s social, emotional, and behavioral functioning. Journal of Applied Developmental Psychology, 61, 21–32. DOI: 10.1016/j.appdev.2018.02.002

[128] Jeon, L., Hur, E., & Buettner, C. K. (2016). Child-care chaos and teachers’ responsiveness: The indirect associations through teachers’ emotion regulation and coping. Journal of School Psychology, 59, 83–96. DOI: 10.1016/j.jsp.2016.09.006 27923443

[129] Jiang, H., Wang, K., Wang, X., Lei, X., & Huang, Z. (2021). Understanding a STEM teacher’s emotions and professional identities: A three-year longitudinal case study. International Journal of STEM Education, 8(1), 1–22. DOI: 10.1186/s40594-021-00309-9

[130] Jiang, J., Vauras, M., Volet, S., & Wang, Y. (2016). Teachers’ emotions and emotion regulation strategies: Self-and students’ perceptions. Teaching and Teacher Education, 54, 22–31. DOI: 10.1016/j.tate.2015.11.008

[131] John-Henderson, N. A., Rheinschmidt, M. L., & Mendoza-Denton, R. (2015). Cytokine responses and math performance: The role of stereotype threat and anxiety reappraisals. Journal of Experimental Social Psychology, 56, 203–206. DOI: 10.1016/j.jesp.2014.10.002

[132] Johnson, C. C. (2012). Implementation of STEM education policy: Challenges, progress, and lessons learned. School science and mathematics, 112(1), 45–55. DOI: 10.1111/j.1949-8594.2011.00110.x

[133] Kaku, M. (2012). Physics of the future: How science will shape human destiny and our daily lives by the year 2100. Anchor.

[134] Katz, D. A., Harris, A., Abenavoli, R., Greenberg, M. T., & Jennings, P. A. (2018). Educators’ emotion regulation strategies and their physiological indicators of chronic stress over 1 year. Stress and Health, 34(2), 278–285. DOI: 10.1002/smi.2782 28990329

[135] Kazén, M., Kuhl, J., & Leicht, E. M. (2015). When the going gets tough…: Self-motivation is associated with invigoration and fun. Psychological Research, 79(6), 1064–1076. DOI: 10.1007/s00426-014-0631-z 25433692

[136] Keller, M. M., & Becker, E. S. (2021). Teachers’ emotions and emotional authenticity: Do they matter to students’ emotional responses in the classroom? Teachers and Teaching, 27(5), 404–422. DOI: 10.1080/13540602.2020.1834380

[137] Keller, M. M., Chang, M. L., Becker, E. S., Goetz, T., & Frenzel, A. C. (2014). Teachers’ emotional experiences and exhaustion as predictors of emotional labor in the classroom: An experience sampling study. Frontiers in Psychology, 5, 1442. DOI: 10.3389/fpsyg.2014.01442 25566124 PMC4263074

[138] Kenworthy, J., Fay, C., Frame, M., & Petree, R. (2014). A meta-analytic review of the relationship between emotional dissonance and emotional exhaustion. Journal of Applied Social Psychology, 44(2), 94–105. DOI: 10.1111/jasp.12211

[139] Kim, C., Kim, D., Yuan, J., Hill, R. B., Doshi, P., & Thai, C. N. (2015). Robotics to promote elementary education pre-service teachers’ STEM engagement, learning, and teaching. Computers & Education, 91, 14–31. DOI: 10.1016/j.compedu.2015.08.005

[140] Klassen, R., & Chiu, M. M. (2010). Effects of teachers’ self-efficacy and job satisfaction: Teacher gender, years of experience, and job stress. Journal of Educational Psychology, 102, 741–756. DOI: 10.1037/a0019237

[141] Kunter, M., Klusmann, U., Baumert, J., Richter, D., Voss, T., & Hachfeld, A. (2013). Professional competence of teachers: Effects on instructional quality and student development. Journal of Educational Psychology, 105(3), 805–820. DOI: 10.1037/a0032583

[142] Kwon, K., Hanrahan, A. R., & Kupzyk, K. A. (2017). Emotional expressivity and emotion regulation: Relation to academic functioning among elementary school children. School Psychology Quarterly, 32(1), 75–88. DOI: 10.1037/spq0000166 27845519

[143] Kwon, K., Kupzyk, K., & Benton, A. (2018). Negative emotionality, emotion regulation, and achievement: Cross-lagged relations and mediation of academic engagement. Learning and Individual Differences, 67, 33–40. DOI: 10.1016/j.lindif.2018.07.004

[144] Lavy, S., & Eshet, R. (2018). Spiral effects of teachers’ emotions and emotion regulation strategies: Evidence from a daily diary study. Teaching and Teacher Education, 73, 151–161. DOI: 10.1016/j.tate.2018.04.001

[145] Layzer Yavin, L., Shechter, A., & Rubinsten, O. (2022). Mathematical and negative information are similarly processed: Pupil dilation as an indicator. Journal of Intelligence, 10(4), 79. DOI: 10.3390/jintelligence10040079 36278601 PMC9624308

[146] Lazarus, R. S. (2001). Relational meaning and discrete emotions. In K. R. Scherer, A. Schorr & T. Johnstone (Eds.), Appraisal processes in emotion: Theory, methods, research (pp. 37–67). Oxford University Press.

[147] Lazarus, R. S., & Folkman, S. (1984). Stress, appraisal, and coping. Springer.

[148] Lee, J. (2009). Universals and specifics of math self-concept, math self-efficacy, and math anxiety across 41 PISA 2003 participating countries. Learning and Individual Differences, 19(3), 355–365. DOI: 10.1016/j.lindif.2008.10.009

[149] Lee, J. C. K., Huang, Y. X. H., Law, E. H. F., & Wang, M. H. (2013). Professional identities and emotions of teachers in the context of curriculum reform: A Chinese perspective. Asia-Pacific Journal of Teacher Education, 41(3), 271–287. DOI: 10.1080/1359866X.2013.809052

[150] Lee, M., Pekrun, R., Taxer, J. L., Schutz, P. A., Vogl, E., & Xie, X. (2016). Teachers’ emotions and emotion management: Integrating emotion regulation theory with emotional labor research. Social Psychology of Education, 19(4), 843–863. DOI: 10.1007/s11218-016-9359-5

[151] Levy-Gigi, E., Bonanno, G. A., Shapiro, A. R., Richter-Levin, G., Kéri, S., & Sheppes, G. (2016). Emotion regulatory flexibility sheds light on the elusive relationship between repeated traumatic exposure and posttraumatic stress disorder symptoms. Clinical Psychological Science, 4(1), 28–39. DOI: 10.1177/2167702615577783

[152] Li, Y., Wang, K., Xiao, Y., & Froyd, J. E. (2020). Research and trends in STEM education: A systematic review of journal publications. International Journal of STEM Education, 7(1), 1–16. DOI: 10.1186/s40594-020-00207-6

[153] Liu, J. J., Ein, N., Gervasio, J., & Vickers, K. (2019). The efficacy of stress reappraisal interventions on stress responsivity: A meta-analysis and systematic review of existing evidence. PLoS One, 14(2), e0212854. DOI: 10.1371/journal.pone.0212854 30811484 PMC6392321

[154] Malmberg, L. E., & Hagger, H. (2009). Changes in student teachers’ agency beliefs during a teacher education year, and relationships with observed classroom quality, and day-to-day experiences. British Journal of Educational Psychology, 79(4), 677–694. DOI: 10.1348/000709909X454814 19558754

[155] Malzahn, K. A. (2013). 2012 National Survey of Science and Mathematics Education: Status of elementary school mathematics. Horizon Research, Inc.

[156] Markus, H. R., & Kitayama, S. (1991). Culture and the self: Implications for cognition, emotion, and motivation. Psychological Review, 98(2), 224–253. DOI: 10.1037/0033-295X.98.2.224

[157] Martin, A. J., & Collie, R. J. (2019). Teacher–student relationships and students’ engagement in high school: Does the number of negative and positive relationships with teachers matter? Journal of Educational Psychology, 111(5), 861–876. DOI: 10.1037/edu0000317

[158] McRae, K., Ciesielski, B., & Gross, J. J. (2012). Unpacking cognitive reappraisal: Goals, tactics, and outcomes. Emotion, 12(2), 250–255. DOI: 10.1037/a0026351 22148990

[159] McRae, K., Jacobs, S. E., Ray, R. D., John, O. P., & Gross, J. J. (2012). Individual differences in reappraisal ability: Links to reappraisal frequency, well-being, and cognitive control. Journal of Research in Personality, 46(1), 2–7. DOI: 10.1016/j.jrp.2011.10.003

[160] Mennin, D. S., & Fresco, D. M. (2009). Emotion regulation as an integrative framework for understanding and treating psychopathology. In A. M. Kring & D. M. Sloan (Eds.), Emotion regulation and psychopathology: A transdiagnostic approach to etiology and treatment (pp. 356–379). Guilford.

[161] Mérida-López, S., Extremera, N., & Rey, L. (2017). Emotion-regulation ability, role stress and teachers’ mental health. Occupational Medicine, 67(7), 540–545. DOI: 10.1093/occmed/kqx125 29016826

[162] Mesquita, B. (2001). Emotions in collectivist and individualist contexts. Journal of Personality and Social Psychology, 80(1), 68–74. DOI: 10.1037/0022-3514.80.1.68 11195892

[163] Moè, A., & Katz, I. (2020). Emotion regulation and need satisfaction shape a motivating teaching style. Teachers and Teaching, 27(5), 1–18. DOI: 10.1080/13540602.2020.1777960

[164] Montgomery, C., & Rupp, A. A. (2005). A meta-analysis for exploring the diverse causes and effects of stress in teachers. Canadian Journal of Education/Revue canadienne de l’éducation, 458–486. DOI: 10.2307/4126479

[165] Muis, K. R., Chevrier, M., & Singh, C. A. (2018). The role of epistemic emotions in personal epistemology and self-regulated learning. Educational Psychologist, 53(3), 165–184. DOI: 10.1080/00461520.2017.1421465

[166] Naghieh, A., Montgomery, P., Bonell, C. P., Thompson, M., & Aber, J. L. (2015). Organisational interventions for improving wellbeing and reducing work-related stress in teachers. Cochrane Database of Systematic Reviews, 4, CD010306. DOI: 10.1002/14651858.CD010306.pub2 PMC10993096 25851427

[167] Näring, G., Briët, M., & Brouwers, A. (2006). Beyond demand–control: Emotional labour and symptoms of burnout in teachers. Work & Stress, 20(4), 303–315. DOI: 10.1080/02678370601065182

[168] National Research Council. (2014). STEM integration in K-12 education: Status, prospects, and an agenda for research. National Academies Press.

[169] Niendam, T. A., Laird, A. R., Ray, K. L., Dean, Y. M., Glahn, D. C., & Carter, C. S. (2012). Meta-analytic evidence for a superordinate cognitive control network subserving diverse executive functions. Cognitive, Affective, & Behavioral Neuroscience, 12(2), 241–268. DOI: 10.3758/s13415-011-0083-5 PMC3660731 22282036

[170] Oberle, E., Gist, A., Cooray, M. S., & Pinto, J. B. (2020). Do students notice stress in teachers? Associations between classroom teacher burnout and students’ perceptions of teacher social–emotional competence. Psychology in the Schools, 57(11), 1741–1756. DOI: 10.1002/pits.22432

[171] Oberle, E., & Schonert-Reichl, K. A. (2016). Stress contagion in the classroom? The link between classroom teacher burnout and morning cortisol in elementary school students. Social Science & Medicine, 159, 30–37. DOI: 10.1016/j.socscimed.2016.04.031 27156042

[172] Ochsner, K. N., Bunge, S. A., Gross, J. J., & Gabrieli, J. D. (2002). Rethinking feelings: An FMRI study of the cognitive regulation of emotion. Journal of Cognitive Neuroscience, 14(8), 1215–1229. DOI: 10.1162/089892902760807212 12495527

[173] Ochsner, K. N., Silvers, J. A., & Buhle, J. T. (2012). Functional imaging studies of emotion regulation: A synthetic review and evolving model of the cognitive control of emotion. Annals of the New York Academy of Sciences, 1251(1), E1–E24. DOI: 10.1111/j.1749-6632.2012.06751.x 23025352 PMC4133790

[174] O’Connor, K. E. (2008). “You choose to care”: Teachers, emotions and professional identity. Teaching and Teacher Education, 24(1), 117–126. DOI: 10.1016/j.tate.2006.11.008

[175] O’Grady, K., Deussing, M. A., Scerbina, T., Tao, Y., Fung, K., Elez, V., & Monk, J. (2018). Measuring up: Canadian results of the OECD PISA 2018 study. The Council of Ministers of Education, Canada (CMEC). https://www.cmec.ca/Publications/Lists/Publications/Attachments/396/PISA2018_PublicReport_EN.pdf

[176] Parr, A., Gladstone, J., Rosenzweig, E., & Wang, M. T. (2021). Why do I teach? A mixed-methods study of in-service teachers’ motivations, autonomy-supportive instruction, and emotions. Teaching and Teacher Education, 98, 103228. DOI: 10.1016/j.tate.2020.103228

[177] Patti, J., Holzer, A. A., Brackett, M. A., & Stern, R. (2015). Twenty-first-century professional development for educators: a coaching approach grounded in emotional intelligence. Coaching: An International Journal of Theory, Research and Practice, 8(2), 96–119. DOI: 10.1080/17521882.2015.1061031

[178] Pekrun, R., Elliot, A. J., & Maier, M. A. (2009). Achievement goals and achievement emotions: Testing a model of their joint relations with academic performance. Journal of Educational Psychology, 101(1), 115–135. DOI: 10.1037/a0013383

[179] Philippot, P., & Brutoux, F. (2008). Induced rumination dampens executive processes in dysphoric young adults. Journal of Behavior Therapy and Experimental Psychiatry, 39(3), 219–227. DOI: 10.1016/j.jbtep.2007.07.001 17698028

[180] Pizzie, R. G., & Kraemer, D. J. (2017). Avoiding math on a rapid timescale: Emotional responsivity and anxious attention in math anxiety. Brain and Cognition, 118, 100–107. DOI: 10.1016/j.bandc.2017.08.004 28826050

[181] Pizzie, R. G., & Kraemer, D. J. (2021). The association between emotion regulation, physiological arousal, and performance in math anxiety. Frontiers in Psychology, 12, 639448. DOI: 10.3389/fpsyg.2021.639448 34045991 PMC8144633

[182] Pizzie, R. G., McDermott, C. L., Salem, T. G., & Kraemer, D. J. (2020). Neural evidence for cognitive reappraisal as a strategy to alleviate the effects of math anxiety. Social Cognitive and Affective Neuroscience, 15(12), 1271–1287. DOI: 10.1093/scan/nsaa161 33258958 PMC7759208

[183] Quinn, M. E., & Joormann, J. (2020). Executive control under stress: Relation to reappraisal ability and depressive symptoms. Behaviour Research and Therapy, 131, 103634. DOI: 10.1016/j.brat.2020.103634 32387887 PMC7336894

[184] Ramachandran, V. (2005). Why school teachers are demotivated and disheartened. Economic and Political Weekly, 40(21), 2141–2144.

[185] Ramirez, G., Hooper, S. Y., Kersting, N. B., Ferguson, R., & Yeager, D. (2018). Teacher math anxiety relates to adolescent students’ math achievement. Aera Open, 4(1), 2332858418756052. DOI: 10.1177/2332858418756052 PMC6502250 31069247

[186] Rege, M., Hanselman, P., Solli, I. F., Dweck, C. S., Ludvigsen, S., Bettinger, E., Crosnoe, R., Muller, C., Walton, G., Duckworth, A., & Yeager, D. S. (2021). How can we inspire nations of learners? An investigation of growth mindset and challenge-seeking in two countries. American Psychologist, 76(5), 755–767. DOI: 10.1037/amp0000647 33180534 PMC8113339

[187] Reinke, W. M., Stormont, M., Herman, K. C., Puri, R., & Goel, N. (2011). Supporting children’s mental health in schools: Teacher perceptions of needs, roles, and barriers. School Psychology Quarterly, 26(1), 1–13. DOI: 10.1037/a0022714

[188] Renninger, K. A., & Hidi, S. E. (2015). The power of interest for motivation and engagement. Routledge. DOI: 10.4324/9781315771045

[189] Richardson, P. W., & Watt, H. M. (2018). Teacher professional identity and career motivation: A lifespan perspective. In P. A. Schultz & J. Hong (Eds.), Research on teacher identity (pp. 37–48). Springer. DOI: 10.1007/978-3-319-93836-3_4

[190] Rodrigo-Ruiz, D. (2016). Effect of teachers’ emotions on their students: Some evidence. Journal of Education & Social Policy, 3(4), 73–79.

[191] Roemer, L., Orsillo, S. M., & Salters-Pedneault, K. (2008). Efficacy of an acceptance-based behavior therapy for generalized anxiety disorder: Evaluation in a randomized controlled trial. Journal of Consulting and Clinical Psychology, 76(6), 1083–1089. DOI: 10.1037/a0012720 19045976 PMC2596727

[192] Roeser, R. W., Skinner, E., Beers, J., & Jennings, P. A. (2012). Mindfulness training and teachers’ professional development: An emerging area of research and practice. Child Development Perspectives, 6(2), 167–173. DOI: 10.1111/j.1750-8606.2012.00238.x

[193] Rottenberg, J., & Gross, J. J. (2003). When emotion goes wrong: Realizing the promise of affective science. Clinical Psychology: Science and Practice, 10(2), 227–232. DOI: 10.1093/clipsy.bpg012

[194] Rozek, C. S., Ramirez, G., Fine, R. D., & Beilock, S. L. (2019). Reducing socioeconomic disparities in the STEM pipeline through student emotion regulation. Proceedings of the National Academy of Sciences, 116(5), 1553–1558. DOI: 10.1073/pnas.1808589116 PMC6358706 30642965

[195] Russo, J., Bobis, J., Sullivan, P., Downton, A., Livy, S., McCormick, M., & Hughes, S. (2020). Exploring the relationship between teacher enjoyment of mathematics, their attitudes towards student struggle and instructional time amongst early years primary teachers. Teaching and Teacher Education, 88, 102983. DOI: 10.1016/j.tate.2019.102983

[196] Sammy, N., Anstiss, P. A., Moore, L. J., Freeman, P., Wilson, M. R., & Vine, S. J. (2017). The effects of arousal reappraisal on stress responses, performance and attention. Anxiety, Stress, & Coping, 30(6), 619–629. DOI: 10.1080/10615806.2017.1330952 28535726

[197] Sandel-Fernandez, D. B., Pearlstein, J. G., Swerdlow, B. A., & Johnson, S. L. (2022). Who disengages from emotion and when? An EMA study of how urgency and distress intolerance relate to daily emotion regulation. Emotion. Advance online publication. DOI: 10.1037/emo0001152 PMC9975112 36048037

[198] Sander, D., Grandjean, D., & Scherer, K. R. (2018). An appraisal-driven componential approach to the emotional brain. Emotion Review, 10(3), 219–231. DOI: 10.1177/1754073918765653

[199] Schaeffer, M. W., Rozek, C. S., Maloney, E. A., Berkowitz, T., Levine, S. C., & Beilock, S. L. (2021). Elementary school teachers’ math anxiety and students’ math learning: A large-scale replication. Developmental Science, 24(4), e13080. DOI: 10.1111/desc.13080 33382186

[200] Schleicher, A. (2019). PISA 2018: Insights and Interpretations. OECD Publishing.

[201] Schönfelder, S., Kanske, P., Heissler, J., & Wessa, M. (2014). Time course of emotion-related responding during distraction and reappraisal. Social Cognitive and Affective Neuroscience, 9(9), 1310–1319. DOI: 10.1093/scan/nst116 23988760 PMC4158366

[202] Schubert, S., Pekrun, R., & Ufer, S. (2023). The role of epistemic emotions in undergraduate students’ proof construction. ZDM–Mathematics Education, 55(2), 299–314. DOI: 10.1007/s11858-022-01413-y

[203] Schukajlow, S., Rakoczy, K., & Pekrun, R. (2017). Emotions and motivation in mathematics education: Theoretical considerations and empirical contributions. ZDM, 49, 307–322. DOI: 10.1007/s11858-017-0864-6 PMC9845103 36684477

[204] Schutz, P. A., Aultman, L. P., & Williams-Johnson, M. R. (2009). Educational psychology perspectives on teachers’ emotions. In P. Schutz & M. Zembylas (Eds.), Advances in teacher emotion research (pp. 195–212). Springer. DOI: 10.1007/978-1-4419-0564-2_10

[205] Schutz, P. A., Hong, J. Y., Cross, D. I., & Osbon, J. N. (2006). Reflections on investigating emotion in educational activity settings. Educational Psychology Review, 18(4), 343–360. DOI: 10.1007/s10648-006-9030-3

[206] Shafir, R., Schwartz, N., Blechert, J., & Sheppes, G. (2015). Emotional intensity influences pre-implementation and implementation of distraction and reappraisal. Social Cognitive and Affective Neuroscience, 10(10), 1329–1337. DOI: 10.1093/scan/nsv022 25700568 PMC4590533

[207] Shafir, R., Thiruchselvam, R., Suri, G., Gross, J. J., & Sheppes, G. (2016). Neural processing of emotional-intensity predicts emotion regulation choice. Social Cognitive and Affective Neuroscience, 11(12), 1863–1871. DOI: 10.1093/scan/nsw114 27522091 PMC5141964

[208] Shansky, R. M., & Lipps, J. (2013). Stress-induced cognitive dysfunction: Hormone-neurotransmitter interactions in the prefrontal cortex. Frontiers in Human Neuroscience, 7, 123. DOI: 10.3389/fnhum.2013.00123 23576971 PMC3617365

[209] Shechter, A., & Share, D. L. (2021). Keeping an eye on effort: A pupillometric investigation of effort and effortlessness in visual word recognition. Psychological Science, 32(1), 80–95. DOI: 10.1177/0956797620958638 33259742 PMC7809337

[210] Sheppes, G. (2020). Transcending the “good & bad” and “here & now” in emotion regulation: Costs and benefits of strategies across regulatory stages. In B. Gawronski (Ed.), Advances in experimental social psychology (Vol. 61, pp. 185–236). Academic Press. DOI: 10.1016/bs.aesp.2019.09.003

[211] Sheppes, G., & Meiran, N. (2008). Divergent cognitive costs for online forms of reappraisal and distraction. Emotion, 8(6), 870–874. DOI: 10.1037/a0013711 19102598

[212] Sheppes, G., Scheibe, S., Suri, G., & Gross, J. J. (2011). Emotion-regulation choice. Psychological Science, 22(11), 1391–1396. DOI: 10.1177/0956797611418350 21960251

[213] Sheppes, G., Scheibe, S., Suri, G., Radu, P., Blechert, J., & Gross, J. J. (2014). Emotion regulation choice: A conceptual framework and supporting evidence. Journal of Experimental Psychology: General, 143(1), 163–181. DOI: 10.1037/a0030831 23163767

[214] Sheppes, G., Suri, G., & Gross, J. J. (2015). Emotion regulation and psychopathology. Annual Review of Clinical Psychology, 11, 379–405. DOI: 10.1146/annurev-clinpsy-032814-112739 25581242

[215] Shernoff, D. J., Sinha, S., Bressler, D. M., & Ginsburg, L. (2017). Assessing teacher education and professional development needs for the implementation of integrated approaches to STEM education. International Journal of STEM Education, 4(1), 1–16. DOI: 10.1186/s40594-017-0068-1 PMC6310389 30631669

[216] Shields, G. S., Sazma, M. A., & Yonelinas, A. P. (2016). The effects of acute stress on core executive functions: A meta-analysis and comparison with cortisol. Neuroscience & Biobehavioral Reviews, 68, 651–668. DOI: 10.1016/j.neubiorev.2016.06.038 27371161 PMC5003767

[217] Skaalvik, E. M. (2018). Mathematics anxiety and coping strategies among middle school students: Relations with students’ achievement goal orientations and level of performance. Social Psychology of Education, 21(3), 709–723. DOI: 10.1007/s11218-018-9433-2

[218] Southward, M. W., & Cheavens, J. S. (2020). More (of the right strategies) is better: Disaggregating the naturalistic between-and within-person structure and effects of emotion regulation strategies. Cognition and Emotion, 34(8), 1729–1736. DOI: 10.1080/02699931.2020.1797637 32696710

[219] Spilt, J. L., Hughes, J. N., Wu, J. Y., & Kwok, O. M. (2012). Dynamics of teacher–student relationships: Stability and change across elementary school and the influence on children’s academic success. Child Development, 83(4), 1180–1195. DOI: 10.1111/j.1467-8624.2012.01761.x 22497209 PMC3399930

[220] Spilt, J. L., Koomen, H. M., & Thijs, J. T. (2011). Teacher wellbeing: The importance of teacher–student relationships. Educational Psychology Review, 23, 457–477. DOI: 10.1007/s10648-011-9170-y

[221] Stark, K., & Bettini, E. (2021). Teachers’ perceptions of emotional display rules in schools: A systematic review. Teaching and Teacher Education, 104, 103388. DOI: 10.1016/j.tate.2021.103388

[222] Stehle, S. M., & Peters-Burton, E. E. (2019). Developing student 21 st Century skills in selected exemplary inclusive STEM high schools. International Journal of STEM Education, 6(1), 1–15. DOI: 10.1186/s40594-019-0192-1

[223] Steiner, E. D., Doan, S., Woo, A., Gittens, A. D., Lawrence, R. A., Berdie, L., Wolfe, R. L., Greer, L., & Schwartz, H. L. (2022). Restoring teacher and principal well-being is an essential step for rebuilding schools: Findings from the State of the American Teacher and State of the American Principal Surveys. RAND Corporation. https://www.rand.org/pubs/research_reports/RRA1108-4.html

[224] Steinhardt, M. A., Smith Jaggars, S. E., Faulk, K. E., & Gloria, C. T. (2011). Chronic work stress and depressive symptoms: Assessing the mediating role of teacher burnout. Stress and Health, 27(5), 420–429. DOI: 10.1002/smi.1394

[225] Stoet, G., Bailey, D. H., Moore, A. M., & Geary, D. C. (2016). Countries with higher levels of gender equality show larger national sex differences in mathematics anxiety and relatively lower parental mathematics valuation for girls. PloS One, 11(4), e0153857. DOI: 10.1371/journal.pone.0153857 27100631 PMC4839696

[226] Sutton, R. E. (2004). Emotional regulation goals and strategies of teachers. Social Psychology of Education, 7(4), 379–398. DOI: 10.1007/s11218-004-4229-y

[227] Sutton, R. E., & Harper, E. (2009). Teachers’ emotion regulation. In L. J. Saha & G. A. Dworkin (Eds.), International handbook of research on teachers and teaching (pp. 389–401). Springer. DOI: 10.1007/978-0-387-73317-3_25

[228] Sutton, R. E., Mudrey-Camino, R., & Knight, C. C. (2009). Teachers’ emotion regulation and classroom management. Theory into Practice, 48(2), 130–137. DOI: 10.1080/00405840902776418

[229] Sutton, R. E., & Wheatley, K. F. (2003). Teachers’ emotions and teaching: A review of the literature and directions for future research. Educational Psychology Review, 15(4), 327–358. DOI: 10.1023/A:1026131715856

[230] Swartz, R. A., & McElwain, N. L. (2012). Preservice teachers’ emotion-related regulation and cognition: Associations with teachers’ responses to children’s emotions in early childhood classrooms. Early Education & Development, 23(2), 202–226. DOI: 10.1080/10409289.2012.619392

[231] Tamir, M. (2016). Why do people regulate their emotions? A taxonomy of motives in emotion regulation. Personality and Social Psychology Review, 20(3), 199–222. DOI: 10.1177/1088868315586325 26015392

[232] Tamir, M., Bigman, Y. E., Rhodes, E., Salerno, J., & Schreier, J. (2015). An expectancy-value model of emotion regulation: Implications for motivation, emotional experience, and decision making. Emotion, 15(1), 90–103. DOI: 10.1037/emo0000021 25198783

[233] Tamir, M., & Millgram, Y. (2017). Motivated emotion regulation: Principles, lessons, and implications of a motivational analysis of emotion regulation. In A. J. Elliot (Ed.), Advances in motivation science, Vol. 4 (pp. 207–247). Elsevier. DOI: 10.1016/bs.adms.2016.12.001

[234] Taxer, J. L., & Frenzel, A. C. (2015). Facets of teachers’ emotional lives: A quantitative investigation of teachers’ genuine, faked, and hidden emotions. Teaching and Teacher Education, 49, 78–88. DOI: 10.1016/j.tate.2015.03.003

[235] Taxer, J. L., & Gross, J. J. (2018). Emotion regulation in teachers: The “why” and “how”. Teaching and Teacher Education, 74, 180–189. DOI: 10.1016/j.tate.2018.05.008

[236] Toh, W. X., & Yang, H. (2022). Common executive function predicts reappraisal ability but not frequency. Journal of Experimental Psychology: General, 151(3), 643. DOI: 10.1037/xge0001099 34472964

[237] Torres, A. S. (2020). Emotions, identity, and commitment among early leavers in the United States of America. Teachers and Teaching, 26(7–8), 508–521. DOI: 10.1080/13540602.2021.1889494

[238] Troy, A. S., Shallcross, A. J., Brunner, A., Friedman, R., & Jones, M. C. (2018). Cognitive reappraisal and acceptance: Effects on emotion, physiology, and perceived cognitive costs. Emotion, 18(1), 58–74. DOI: 10.1037/emo0000371 29154585 PMC6188704

[239] Troy, A. S., Shallcross, A. J., & Mauss, I. B. (2013). A person-by-situation approach to emotion regulation: Cognitive reappraisal can either help or hurt, depending on the context. Psychological Science, 24(12), 2505–2514. DOI: 10.1177/0956797613496434 24145331

[240] Troy, A. S., Willroth, E. C., Shallcross, A. J., Giuliani, N. R., Gross, J. J., & Mauss, I. B. (2022). Psychological resilience: An affect-regulation framework. Annual Review of Psychology, 74, 18.1–18.30. DOI: 10.1146/annurev-psych-020122-041854 PMC12009612 36103999

[241] Tsang, K. K., & Jiang, L. (2018). Positive emotional experiences in teaching, teacher identity, and student behaviors: A symbolic interactionist perspective. Schools, 15(2), 228–246. DOI: 10.1086/699890

[242] Tsang, K. K., & Kwong, T. L. (2017). Teachers’ emotions in the context of education reform: Labor process theory and social constructionism. British Journal of Sociology of Education, 38(6), 841–855. DOI: 10.1080/01425692.2016.1182007

[243] Tsouloupas, C. N., Carson, R. L., Matthews, R., Grawitch, M. J., & Barber, L. K. (2010). Exploring the association between teachers’ perceived student misbehaviour and emotional exhaustion: The importance of teacher efficacy beliefs and emotion regulation. Educational Psychology, 30(2), 173–189. DOI: 10.1080/01443410903494460

[244] Uitto, M., Jokikokko, K., & Estola, E. (2015). Virtual special issue on teachers and emotions in Teaching and teacher education (TATE) in 1985–2014. Teaching and Teacher Education, 50, 124–135. DOI: 10.1016/j.tate.2015.05.008

[245] Uribe-Mariño, A., Gassen, N. C., Wiesbeck, M. F., Balsevich, G., Santarelli, S., Solfrank, B., Dournes, C., Fries, G. R., Masana, M., Labermeier, C., Wang, X. D., Hafner, K., Schmid, B., Rein, T., Chen, A., Deussing, J. M., & Schmidt, M. V. (2016). Prefrontal cortex corticotropin-releasing factor receptor 1 conveys acute stress-induced executive dysfunction. Biological Psychiatry, 80(10), 743–753. DOI: 10.1016/j.biopsych.2016.03.2106 27318500

[246] Uzuntiryaki-Kondakci, E., Kirbulut, Z. D., Oktay, O., & Sarici, E. (2022). A qualitative examination of science teachers’ emotions, emotion regulation goals and strategies. Research in Science Education, 52(4), 1131–1155. DOI: 10.1007/s11165-020-09986-y

[247] van der Want, A. C., den Brok, P., Beijaard, D., Brekelmans, M., Claessens, L. C., & Pennings, H. J. (2015). Teachers’ interpersonal role identity. Scandinavian Journal of Educational Research, 59(4), 424–442. DOI: 10.1080/00313831.2014.904428

[248] Veldman, I., Van Tartwijk, J., Brekelmans, M., & Wubbels, T. (2013). Job satisfaction and teacher–student relationships across the teaching career: Four case studies. Teaching and Teacher Education, 32, 55–65. DOI: 10.1016/j.tate.2013.01.005

[249] Vitolo, E., Diano, M., Giromini, L., & Zennaro, A. (2022). Markers of emotion regulation processes: A neuroimaging and behavioral study of reappraising abilities. Biological Psychology, 171, 108349. DOI: 10.1016/j.biopsycho.2022.108349 35569572

[250] Vogel, S., Fernández, G., Joëls, M., & Schwabe, L. (2016). Cognitive adaptation under stress: a case for the mineralocorticoid receptor. Trends in Cognitive Sciences, 20(3), 192–203. DOI: 10.1016/j.tics.2015.12.003 26803208

[251] Wang, K., Chen, Z., Luo, W., Li, Y., & Waxman, H. (2018). Examining the differences between the job satisfaction of STEM and non-STEM novice teachers with leaving intentions. EURASIA Journal of Mathematics, Science and Technology Education, 14(6), 2329–2341. DOI: 10.29333/ejmste/89516

[252] Wante, L., Mezulis, A., Van Beveren, M. L., & Braet, C. (2017). The mediating effect of adaptive and maladaptive emotion regulation strategies on executive functioning impairment and depressive symptoms among adolescents. Child Neuropsychology, 23(8), 935–953. DOI: 10.1080/09297049.2016.1212986 27535347

[253] Whitaker, R. C., Dearth-Wesley, T., & Gooze, R. A. (2015). Workplace stress and the quality of teacher–children relationships in head start. Early Childhood Research Quarterly, 30(Part A), 57–69. DOI: 10.1016/j.ecresq.2014.08.008

[254] Wilms, R., Lanwehr, R., & Kastenmüller, A. (2020). Emotion regulation in everyday life: The role of goals and situational factors. Frontiers in Psychology, 877. DOI: 10.3389/fpsyg.2020.00877 32508713 PMC7248400

[255] Winne, P. H. (2019). Paradigmatic dimensions of instrumentation and analytic methods in research on self-regulated learning. Computers in Human Behavior, 96, 285–289. DOI: 10.1016/j.chb.2019.03.026

[256] Wong, V. W., Ruble, L. A., Yu, Y., & McGrew, J. H. (2017). Too stressed to teach? Teaching quality, student engagement, and IEP outcomes. Exceptional Children, 83(4), 412–427. DOI: 10.1177/0014402917690729 30555178 PMC6294446

[257] Xu, J., Du, J., Liu, F., & Huang, B. (2019). Emotion regulation, homework completion, and math achievement: Testing models of reciprocal effects. Contemporary Educational Psychology, 59, 101810. DOI: 10.1016/j.cedpsych.2019.101810

[258] Yan, E. M., Evans, I. M., & Harvey, S. T. (2011). Observing emotional interactions between teachers and students in elementary school classrooms. Journal of Research in Childhood Education, 25(1), 82–97. DOI: 10.1080/02568543.2011.533115

[259] Yeager, D. S., Bryan, C. J., Gross, J. J., Murray, J. S., Krettek Cobb, D., HF Santos, P., Gravelding, H., Johnson, M., & Jamieson, J. P. (2022). A synergistic mindsets intervention protects adolescents from stress. Nature, 607(7919), 512–520. DOI: 10.1038/s41586-022-04907-7 35794485 PMC9258473

[260] Yeager, D. S., Hanselman, P., Walton, G. M., Murray, J. S., Crosnoe, R., Muller, C., Tipton, E., Schneider, B., Hulleman, C. S., Hinojosa, C. P., Paunesku, D., Romero, C., Flint, K., Roberts, A., Trott, J., Iachan, R., Buontempo, J., Yang, S. M., Carvalho, C. M., Hahn, P. R., Gopalan, M., Mhatre, P., Ferguson, R., Duckworth, A. L., & Dweck, C. S. (2019). A national experiment reveals where a growth mindset improves achievement. Nature, 573, 364–369. DOI: 10.1038/s41586-019-1466-y 31391586 PMC6786290

[261] Yeager, D. S., & Dweck, C. S. (2020). What can be learned from growth mindset controversies?. American Psychologist, 75(9), 1269–1284. DOI: 10.1037/amp0000794 33382294 PMC8299535

[262] Yeager, D. S., Romero, C., Paunesku, D., Hulleman, C. S., Schneider, B., Hinojosa, C., Lee, H. Y., O’Brien, J., Flint, K., Roberts, A., Trott, J., Greene, D., Walton, G. M., & Dweck, C. S. (2016). Using design thinking to improve psychological interventions: The case of the growth mindset during the transition to high school. Journal of Educational Psychology, 108(3), 374–391. DOI: 10.1037/edu0000098 27524832 PMC4981081

[263] Yih, J., Uusberg, A., Taxer, J. L., & Gross, J. J. (2019). Better together: A unified perspective on appraisal and emotion regulation. Cognition and Emotion, 33(1), 41–47. DOI: 10.1080/02699931.2018.1504749 30058449

[264] Yin, H., Huang, S., & Lv, L. (2018). A multilevel analysis of job characteristics, emotion regulation, and teacher well-being: A job demands-resources model. Frontiers in Psychology, 9, 2395. DOI: 10.3389/fpsyg.2018.02395 30555395 PMC6281830

[265] Zaki, J., & Williams, W. C. (2013). Interpersonal emotion regulation. Emotion, 13(5), 803–810. DOI: 10.1037/a0033839 24098929

[266] Zembylas, M. (2002). Constructing genealogies of teachers’ emotions in science teaching. Journal of Research in Science Teaching: The Official Journal of the National Association for Research in Science Teaching, 39(1), 79–103. DOI: 10.1002/tea.10010

[267] Zinsser, K. M., Bailey, C. S., Curby, T. W., Denham, S. A., & Bassett, H. H. (2013). Exploring the predictable classroom: Preschool teacher stress, emotional supportiveness, and students’ social-emotional behavior in private and Head Start classrooms. HS Dialog: The Research to Practice Journal for the Early Childhood Field, 16(2), 90–108.

[268] Zinsser, K. M., Denham, S. A., & Curby, T. W. (2018). Becoming a Social and Emotional Teacher The Heart of Good Guidance. YC Young Children, 73(4), 77–83.

